# SCONE: a subset-contrastive method for multi-omics network embedding

**DOI:** 10.1093/bib/bbag381

**Published:** 2026-08-12

**Authors:** Pedro Henrique da Costa Avelar, Jonathan Cardoso-Silva, Min Wu, Sophia Tsoka

**Affiliations:** Department of Informatics, Faculty of Natural, Mathematical and Engineering Sciences, King’s College London, Bush House, 30 Aldwych, London WC2B 4BG, United Kingdom; Institute for Advanced Intelligence and Computing, Agency for Science Technology and Research (A^*^STAR), 1 Fusionopolis Way, #21-01 Connexis, 138632, Singapore; Division of Informatics, Imaging and Data Science, Faculty of Biology, Medicine and Health, University of Manchester, Oxford Road, Manchester M13 9PL, United Kingdom; The Data Science Institute, London School of Economics and Political Science, Columbia House, 69 Aldwych, London WC2B 4RR, United Kingdom; Institute for Advanced Intelligence and Computing, Agency for Science Technology and Research (A^*^STAR), 1 Fusionopolis Way, #21-01 Connexis, 138632, Singapore; Department of Informatics, Faculty of Natural, Mathematical and Engineering Sciences, King’s College London, Bush House, 30 Aldwych, London WC2B 4BG, United Kingdom

**Keywords:** graph contrastive learning, single-cell, multi-omics, autoencoder

## Abstract

Network-based analyses of omics data are widely used and, while many of these methods have been adapted to single-cell scenarios, they often remain memory- and space-intensive. As a result, they are better suited to batch data or smaller datasets. Furthermore, the application of network-based methods in multi-omics often relies on similarity-based networks, which lack structurally discrete topologies. This limitation may reduce the effectiveness of graph-based methods that were initially designed for topologies with better defined structures. We propose Subset-Contrastive multi-Omics Network Embedding (SCONE), a method that employs contrastive learning techniques on large datasets through a scalable subgraph contrastive approach. By exploiting the pairwise similarity basis of many network-based omics methods, we transformed this characteristic into a strength, developing an approach that aims to achieve scalable and effective analysis. Our method demonstrates synergistic omics integration for cell type clustering in single-cell data. Additionally, we evaluate its performance in a bulk multi-omics integration scenario, where SCONE performs comparable with the state-of-the-art despite utilizing limited views of the original data. We anticipate that our findings will motivate further research into the use of subset contrastive methods for omics data.

## Introduction

Precision medicine increasingly depends on the ability to integrate molecular information across multiple omic layers, capturing the complex interplay of genomic, transcriptomic, epigenomic, and proteomic regulation that underlies disease phenotypes [1, 2]. Recent advances in single-cell and spatial multi-omics technologies now allow such measurements at unprecedented scale and resolution [3]. These developments have created both an opportunity and a challenge, i.e. the opportunity to map biological systems comprehensively, and the challenge of developing analytical frameworks that can extract meaningful structure from vast, heterogeneous datasets.

Network-based analyses have long been recognized as powerful means to uncover relationships between network topology and system behaviour [4–7], and have been widely applied to the integration of single- and multi-omics data across batch, single-cell, and spatial omics contexts [8]. Representing omics measurements as graphs enables the identification of modules, regulatory circuits, and functional associations that would otherwise be difficult to infer from isolated data types.

Recent advances in machine learning, particularly in deep learning, have significantly expanded the potential of network-based methods, with graph [9, 10] and knowledge graph embedding techniques [11–13] becoming integral to the standard toolkit for biological analyses [6, 7]. Building on these foundations, graph contrastive learning (GCL) methods have emerged as a powerful paradigm for exploiting structural information in biological networks, allowing models to learn representations that are both discriminative and topology-aware [14–16]. These methods learn low-dimensional representations that preserve topological and semantic information, providing a principled way to integrate multi-modal biological data.

Despite their promise, the application of network analysis in omics data often relies on similarity-derived graphs (k-nearest neighbours (KNN) or correlation networks) which lack well-defined discrete topologies. Such approximate structures limit the effectiveness of many GCL algorithms that were originally designed for explicit edge relationships. Moreover, existing graph contrastive models typically require pairwise comparisons across all nodes, resulting in quadratic memory growth that becomes prohibitive for large single-cell or spatial datasets.

To address these limitations, we propose SCONE (Subset-Contrastive multi-Omics Network Embedding) a scalable framework that adapts subset-contrastive learning to network-based multi-omics integration. SCONE transforms the pairwise similarity foundation of omics graphs into an advantage, learning robust joint embeddings from overlapping data subsets while maintaining linear-to-sublinear memory complexity. Through comparative analysis with state-of-the-art methods, we demonstrate that SCONE achieves competitive performance in both bulk and single-cell multi-omics scenarios while substantially reducing computational cost. More broadly, this work highlights the potential of subset-based contrastive learning as a general strategy for scalable biological network analysis.

## Related work

Multi-omics representation learning has become a cornerstone of integrative biomedical analysis, providing a means to uncover latent biological relationships and enhance downstream predictive modelling. Early approaches such as MOFA and MOFA+ established probabilistic frameworks for multi-view data integration [17, 18], and subsequent studies extended these ideas using product-of-experts (PoE) and variational information bottleneck formulations to model complex cross-omic dependencies in bulk datasets [19].

Beyond probabilistic models, generative and contrastive learning methods have gained prominence for multi-modal integration. Subtype-GAN introduced a generative adversarial network (GAN) for joint omics representation [20], while methods such as Subtype-DCC, DMCL, and MOCSS adapted contrastive objectives to multi-view datasets to improve clustering and subtype discovery [21–23]. These methods highlighted the potential of contrastive and generative approaches for aligning heterogeneous omics modalities.

Recently, graph neural components have been incorporated into multi-omics integration frameworks, motivated by the view that biological systems can be naturally represented as networks. Subtype-MGTP employs graph convolutional modules to embed omic features within a multi-omics-to-protein translation framework, producing predicted protein expression profiles that are subsequently clustered using a contrastive deep subspace approach [24]. Subtype-MGTP jointly analyses multiple omics modalities for protein translation, with clustering performed on the inferred protein representation rather than a multi-omics latent representation. While this approach leverages graph neural modules to encode omic structure, it does not explicitly operate on sample-level similarity graphs or perform GCL.

The emergence of high-dimensional single-cell and spatial multi-omics technologies [3] has spurred the development of scalable methods such as TotalVI [25] and Multigrate [26] which combine probabilistic modelling with nonlinear data integration. Among recent graph-based approaches, GraphST [27] constructs KNN graphs from spatial transcriptomic profiles and applies Deep Graph Infomax (DGI) [28] to learn embeddings that preserve spatial structure and improve clustering performance. Similarly, scZAG [29] incorporated a zero-inflated negative binomial variational autoencoder [30] alongside GCL to improve single-cell clustering accuracy.

Recent graph neural network-based frameworks have also been proposed for spatial multi-omics integration, including SpatialGlue [31] and PRAGA [32], which leverage spatial neighbourhood information to align multiple molecular modalities across tissue sections. While these methods demonstrate strong performance in spatial transcriptomics settings, they are specifically designed for spatially resolved data and rely on deterministic, full-graph message passing. By contrast, non-spatial single-cell and bulk multi-omics settings require learning from similarity-derived graphs rather than physical neighbourhoods, motivating scalable objectives that can operate on sampled subgraphs and adapt to modality-specific biological signal.

Despite these advances, most GCL frameworks applied in omics data rely on similarity-derived graph structures (typically k-nearest-neighbour or correlation networks), which lack the discrete topologies that many GCL methods were designed to exploit. As a result, algorithms originally designed for discrete graphs may perform suboptimally, and their pairwise contrastive objectives often entail quadratic memory costs, limiting scalability to large single-cell datasets [15, 29].

To address these limitations, subset-contrastive learning has recently emerged in the machine learning literature as an effective strategy for scalable graph representation learning. Methods such as SubG-Con [33] and AdaGCL [34] achieve comparable embedding performance to full-graph approaches while reducing computational overhead by training on overlapping node subsets. However, these techniques have not yet been adapted to biological data. SCONE adapts these techniques to address this gap by adapting subset-contrastive principles to multi-omics integration. It leverages the similarity-based nature of omics graphs to enable efficient, subgraph-level contrastive training, achieving near-linear scalability while preserving biological structure across modalities.

## Materials

To evaluate SCONE, we used both single-cell and bulk multi-omics datasets representing complementary biological contexts and data modalities. The first dataset comprises peripheral blood mononuclear cells (PBMCs) profiled by single-cell RNA sequencing (scRNA-seq) and surface protein quantification via CITE-seq as described [35]. Cell-type annotations were derived from CITE-seq clustering and validated through corresponding RNA-seq expression. Quality control was performed according to Lotfollahi *et al*. [26], with RNA-seq data normalized to a total count of 10 000 and transformed using $\log{(x+1)}$. Protein counts were normalized using the centred log-ratio transformation [36]. Data were z-scored to mean of 0 and a SD of 1 before being input to neural networks. After preprocessing, the dataset contained 52 117 cells from 20 samples of high and low responders. Cell types were clustered and annotated based on surface protein information, yielding 10 broad cell types at the first level of annotation (see Table 1).

**Table 1:** Cell type composition of the PBMC dataset from [35].

Cell type	Count	Proportion (%)
Total	52 117	*100.00*
CD4 naive	11 127	*21.35*
CD4 memory T	9161	*17.58*
Classical monocytes and mDC	6631	*12.73*
B	5,810	*11.15*
CD8 memory T	5268	*10.11*
CD8 naive	4840	*9.29*
NK	4,700	*9.02*
Unconventional T cells	3268	*6.27*
Non-classical monocytes	1072	*2.06*
pDC	240	*0.46*

The second dataset is derived from The Cancer Genome Atlas (TCGA) and has been preprocessed previously [20, 24]. It comprises five omics layers: bulk RNA expression (rna), miRNA (miRNA), methylation (meth), copy number (CN), and protein (prot); spanning seven cancer types: bladder cancer (BLCA), breast cancer (BRCA), kidney clear cell cancer (KIRC), lung adenocarcinoma (LUAD), skin cutaneous melanoma (SKCM), stomach adenocarcinoma (STAD), and uterine corpus endometrial carcinoma (UCEC). The RNA, miRNA, methylation, and copy number layers were preprocessed according to Yang *et al*. [20], while the protein layer was obtained by Xie *et al*. [24]. In total, these subdatasets comprised 3013 samples with details in Table 2.

**Table 2:** Composition of the TCGA dataset for bulk analysis (age is mean *±* SD, number of clusters derived from Yang *et al*. [20] and Xie *et al*. [24]).

Cancer	Samples	Male/Female (%)	Age	Clusters
BLCA	331	74.92/25.08	*67.88± 10.63*	5
BRCA	829	^a^1.09/98.79	*58.22± 13.39*	5
KIRC	438	65.98/34.02	*60.38± 12.07*	4
LUAD	346	46.53/53.47	*64.57± 9.86*	3
SKCM	333	59.46/40.54	*58.13± 15.79*	4
STAD	329	66.57/33.43	*65.41± 11.01*	3
UCEC	407	0.00/100.0	*63.64± 11.26*	4

^a^Gender totals do not sum to 100% due to missing values.

Although Uveal Melanoma (UVM) was included in datasets used by Yang *et al*. [20] and Xie *et al*. [24], it was excluded here due to its limited sample size (*n=12* for the CN and protein layers), which was insufficient for reliable model training and evaluation. Furthermore, even the strongest baseline models performed poorly on this dataset, likely due to its small scale relative to the other cohorts, so its exclusion is not expected to impact the overall conclusions of this work. In contrast to [24], each cancer type was treated independently, with no information shared across datasets. To ensure consistent subtyping evaluation, only samples containing all five omics layers were retained and samples missing information were excluded during downstream processing.

## Methodology

In order to outline the methodology employed in our experiments, first an overview of our framework is described, followed by detailed description of the graph attention network (GAT) layer, the framework of our proposed model, and finally the experimental pipelines and performance metrics utilized. Additional experimental details can be found in Supplementary Material for “SCONE: Subset-Contrastive Multi-Omics Network Embedding,” Experimental Setup section.

### Overall framework

SCONE integrates multiple omic modalities through a unified graph-based embedding framework (Fig. 1). The model is implemented as a multi-modal graph autoencoder trained using a subset-contrastive learning objective, where modality-specific graph encoders are combined through a Product-of-Experts fusion mechanism. Each omic dataset is represented as a similarity graph in which nodes correspond to samples (cells or patients) and edges encode molecular similarity. Modality-specific graph encoders learn latent embeddings that capture local and global structure within each omic layer.

**Figure 1.**
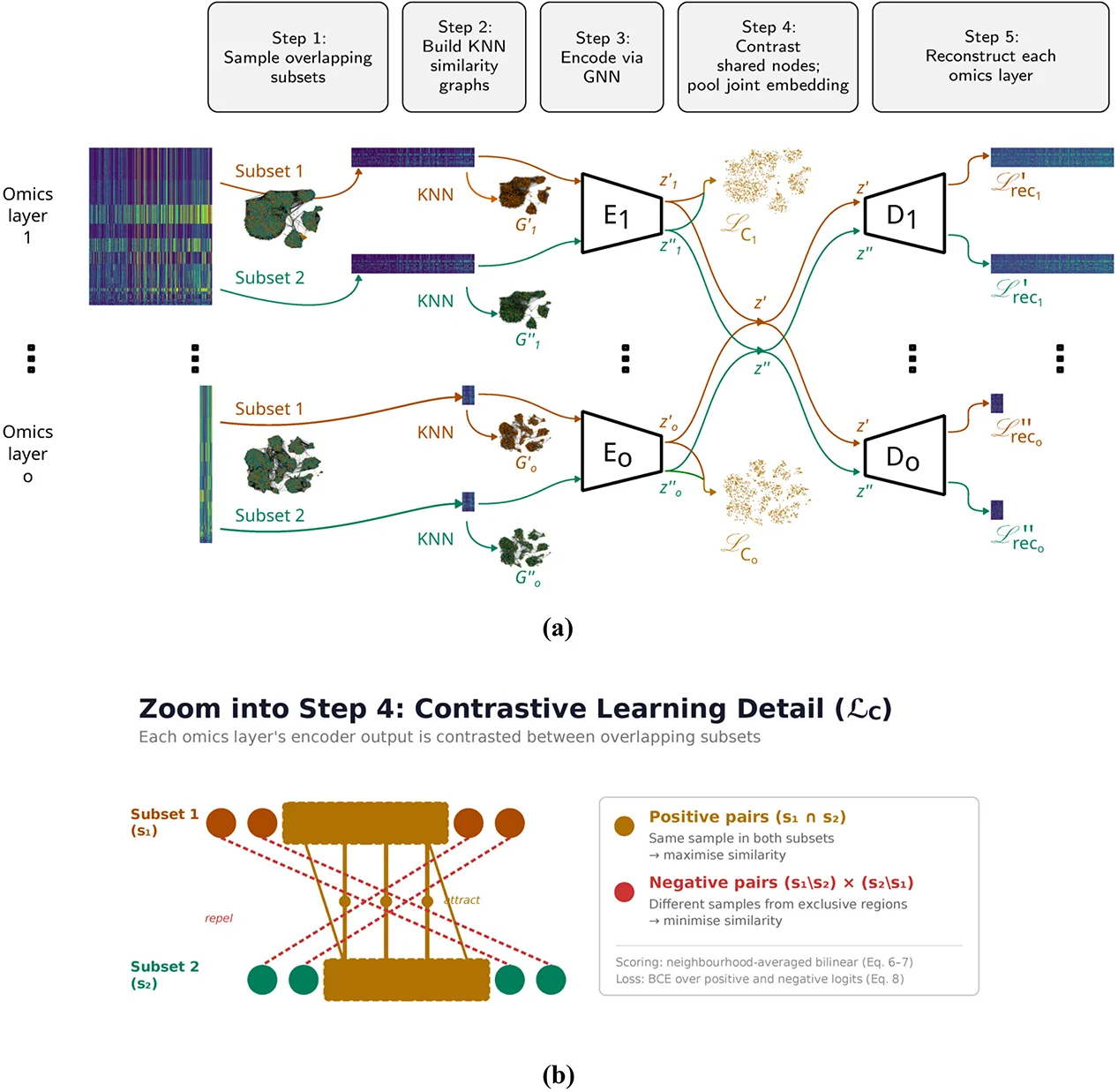
(a) A schematic representation of the proposed SCONE model. See Section Overall framework for a description. The heatmaps shown correspond to the [35] dataset, with omics layer 1 being the RNA data and Omics layer 0 being CITE-seq data. For readability, we reduce the number of samples, RNA probes, and CITE-seq probes by a factor of *∼ *101, 10, and 2, respectively, with subsets further reducing the number of samples by *∼ *10. During inference, the trained encoders $E_{1} \dots E_{o}$ are applied to the full dataset without subsetting, and per-omics embeddings are pooled into a joint representation $z \in \mathbb{R}^{n \times d_{z}}$ (one vector per sample) for downstream analysis. No contrastive loss or reconstruction is computed at inference. (b) An inset showing the details of how the contrastive learning works, where we analyse the neighbourhood average of each node, and negative pairs are randomly assigned.

The model begins by subsetting the original dataset, randomly sampling $k_{s}$ samples from each omics layer to form two overlapping subsets, illustrated as subset 1 and subset 2 in Fig. 1. For each subset, a KNN graph is constructed for each omics layer to capture local sample relationships. The omics features and corresponding graph of each subset are then input into an omics-specific GAT encoder $E_{i}$ to yield the latent embeddings $z_{i} = E_{i}(x_{i}, G_{i})$. Here, the index $i \in \{1,\dots ,|O|\}$ denotes the omics layer (e.g. RNA, protein, and methylation), $x_{i} \in \mathbb{R}^{n_{i} \times d_{i}}$ is the feature matrix for omics layer *i*, and $G_{i} = (V_{i}, E_{i})$ is the corresponding KNN similarity graph whose nodes represent samples and edges encode pairwise similarity within that omics layer. Subset-specific embeddings are denoted as $z^{\prime }{i}$ for subset 1 and $z^{\prime \prime }{i}$ for subset 2. These embeddings are integrated into a shared representation *z*, which is subsequently passed through the omics-specific GAT decoder $D_{i}$ to reconstruct each omic layer for each subset. Reconstruction quality is optimized via the loss $\mathscr{L}_{\mathrm{rec}_{i}}$. Simultaneously, the overlapping nodes (yellow in Fig. 1) between subsets are leveraged for the contrastive loss $\mathscr{L}_{C_{i}}$, which utilizes the encoder outputs and neighbourhood information for both overlapping and nonoverlapping nodes. In Fig. 1, red and green denote the independent trajectories of subsets 1 and 2, respectively, and yellow indicates their overlap which is used to compute the subset-contrastive objective.

### Graph attention networks

In our demonstration of the SCONE framework, we use a variant of GATs with *dynamic attention* [37]. GATs [38] are a graph neural network architecture that learn to aggregate information from a node neighbourhood through attention mechanisms. They consist of multiple graph attention layers, each of which processes an input matrix $h^{l-1} \in R^{n \times d_{l-1}}$ and a graph *G = (V,E)* to produce an output matrix $h^{l} \in R^{n \times d_{l}}$. Attention factors $\alpha _{i,j}$ are computed between a node *i ∈ V* and its neighbours $j \in \mathscr{N}(i)$, where $\mathscr{N}(i)$ is the neighbourhood of *i* defined by the edge list *E*. Specifically, $\exists \alpha _{i,j} \iff \exists (j,i) \in E$. These factors are typically computed using Equation (1), where $e:\mathbb{R}^{d_{l-1}} \times \mathbb{R}^{d_{l-1}} \rightarrow \mathbb{R}^{1}$ is a function that quantifies the similarity between two input vectors. Note that in this section we follow standard graph neural network notation, where indices *i* and *j* refer to nodes (samples) within a graph; this is distinct from what is presented in the Overall Framework section, where the index *i* was used to denote omics layers.


(1) \begin{align*}& \alpha_{i,j} = \frac{\exp{(e(h^{l-1}_{i},h^{l-1}_{j})}}{\sum_{k \in \mathscr{N}(i)}{\exp{(e(h^{l-1}_{i},h^{l-1}_{k})}}}.\end{align*}

These attention factors weight the neighbourhood aggregation function, enabling the computation of a node’s updated representation as a weighted sum of its neighbours’ features, transformed by a nonlinear layer with weight $W_{l}$ and nonlinearity *σ *, as shown in Equation (2): 


(2) \begin{align*}& \begin{aligned} h^{l} &= \operatorname{GAT}(h^{l-1},G) \\ \text{where } h^{l}_{i} &= \sigma\left(\sum_{j \in \mathscr{N}(i)} \alpha_{i,j} h^{l-1,\prime}_{j}\right) \\ \text{and } h^{l-1,\prime} &= h^{l-1} \times W_{l}. \end{aligned}\end{align*}

In the original GAT implementation [38], the attention factor is defined as in Equation (3), where $W \in \mathbb{R}^{d_{h}\times d_{h}^{\prime }}$, and $a \in \mathbb{R}^{2d^{\prime } \times 1}$ are the learnable parameters, and ∥ denotes concatenation along the feature dimension: 


(3) \begin{align*}& e(h_{i},h_{j}) = \operatorname{LeakyReLU}([(h_{i}\times W)\|(h_{j}\times W)] \times a).\end{align*}

However, it has been demonstrated that the attention mechanism originally developed for GAT employs a *static attention* mechanism. This design limits its adaptability, as it cannot attend to different keys (i.e. vectors indexed by *j*, representing the neighbours of a node) when provided with varying queries (i.e. vectors indexed by *i*, representing the targets of the edges). To address this limitation, we employ the $\operatorname{GAT}$ operator implemented through the GATv2Conv module [37] in the pytorch-geometric library [39] (version 2.5.2). This operator introduces a *dynamic attention* mechanism, defined in Equation (4), where a learned linear transformation $W \in \mathbb{R}^{2d \times d^{\prime }}$ is applied after concatenation, followed by a nonlinearity and another linear layer $a \in \mathbb{R}^{d \times 1}$. This approach effectively employs a multilayer perceptron, which is a universal function approximator [40], to compute the attention scores: 


(4) \begin{align*}& e(h_{i},h_{j}) = \operatorname{LeakyReLU}([(h_{i}\|h_{j}W) \times W]) \times a.\end{align*}

### Subset-Contrastive multi-Omics Network Embedding framework

Building on the modality-specific graph embeddings produced by the encoders described in the previous section, SCONE was developed by adapting a Gaussian-prior Product of Experts (PoE) [41] Variational Autoencoder (VAE) [19] into a Multi-modal Deep Autoencoder [42]. Unlike [42], SCONE does not rely on pretraining using restricted Boltzmann machines. Instead, all pretraining is performed through contrastive learning inspired by DGI [28]. The encoder and decoder architectures employ graph-based modules, specifically using GATs [37, 38]. A detailed description of the framework is provided below and visualized in Fig. 1.

The SCONE framework handles $\left |O\right |$ Omics views, where each omics view 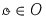 contains a data table 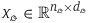 with 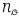 samples 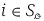 (e.g. single cells or bulk samples) and 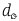 features (e.g. gene or protein expression probes). The framework’s goal is to construct individual latent spaces 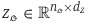 for each omics view, which are then combined into a common latent space $z \in \mathbb{R}^{n \times d_{z}}$. From *z*, the model reconstructs each input table 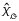 and then applies a reconstruction loss 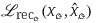 for each omics layer.

To achieve this, omics-specific encoders 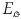 are constructed using GATs. As GATs require a graph as input and omics data do not inherently form a network, a KNN-graph 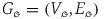 is generated for each omic layer, connecting each sample 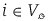 to its $k_{k}$ nearest neighbours. Unless otherwise stated, we use *k*=15 for graph construction, following common practice in previous work [43] and supported by sensitivity analysis (Fig. S8). Each omics measurement produces a hidden latent space as described in Equation (5). 


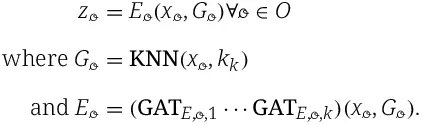
 (5)

These individual latent spaces 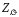 are combined into a joint latent space *z* through a quantity- and order-invariant pooling operation *p*, as shown in Equation (6). Considering that each 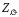 is an element in an Abelian group and *p(a,b)* being the operation, we can then treat missing values by substituting them with the group’s identity $\mathbb{I}$. Without loss of generality, we consider *p* to be the sum between vectors, and use an empty array of zeros $\mathbb{I} \in \mathbb{R}^{d_{z}}, \mathbb{I}[j] = 0 \forall j$. This adaptation using an Abelian group, alongside other properties that we discuss below, allows us to adapt the PoE framework into a nonvariational model [19, 41]. 


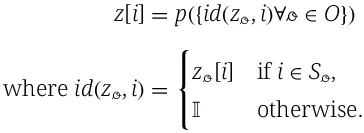
 (6)

From *z*, each omics layer is reconstructed using omics-specific graph decoders 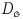, which also utilize GATs. Reconstruction is described in Equation (7): 


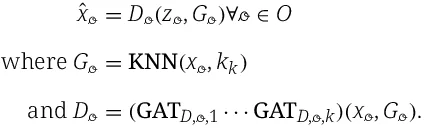
 (7)

The reconstruction loss 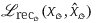 is applied to each of the reconstructed output where ground-truth is available. This allows the network to reconstruct each omics layer from its available data from a joint latent space which might not contain all information. For normally distributed input, the mean-squared error is used (Equation (8)). However, we have also implemented in our software losses for Negative Binomial (NB), Zero-Inflated Negative Binomial (ZINB) and Binary inputs (which can be seen in Equation (9), where we have a probability matrix 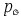 instead of 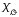). These reconstruction functions are commonplace in omics data (e.g. single-cell RNA measurements are commonly represented through an NB/ZINB distribution [25, 30, 44], and methylation beta-values can be interpreted as binary values or probabilities [45]). 


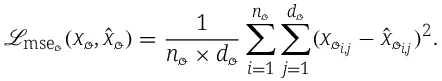
 (8)


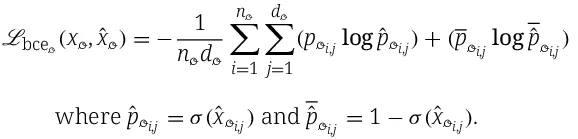
 (9)

However, using the full input matrix and induced connectivity graph may be computationally infeasible, particularly during training, when the number of samples is large, as is often the case in single-cell multi-omics data. To address this, we propose our subset-contrastive framework. Instead of using the full omics input matrix 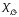, we subset each omics matrix into two overlapping subsets of samples, $s_{1}$ and $s_{2}$, during each training epoch. These subsets are defined such that 
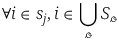
, $s_{1} \cap s_{2} \neq s_{1}$, $\left |s_{1} \cup s_{2}\right |> k_{s}$. This approach ensures that $s_{1}$ and $s_{2}$ share some samples but are not identical.

Using these subsets, we construct two versions of the omics input matrices: 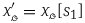 and 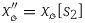. For each subset, we generate corresponding KNN graphs 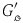 and 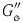, resulting in two sets of latent representations of the omics data: $z^{\prime }$ and 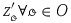 for the first subset and $z^{\prime \prime }$ and 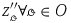 for the second subset.

Inspired by DGI [28], we define a per-omics layer subset-contrastive loss 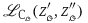 as the Binary Cross Entropy of the similarity of the neighbourhood average of positive and negative examples. By applying this contrastive loss to each individual omics layer representation rather than the joint latent space, the network can learn complementary information about each omics layer independently, which is then aggregated into the joint representation.

Positive examples $s_{+}$ are defined as samples *i* that appear in both subsets, $i \in (s_{1} \cup s_{2})$. Negative examples are defined separately for each subset: $s_{-,1}$ includes samples $i \in (s_{1} \setminus s_{2})$ and $s_{-,2}$ includes samples $j \in (s_{2} \setminus s_{1})$. to compute probabilities for positive and negative pairs, as shown in Equation (10). Here *† * and *‡ * represent either *\prime * or $\prime \prime $, depending on the subset being processed. 


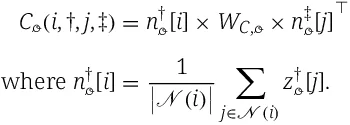
 (10)

The contrastive loss is then computed as the BCE loss between the logits of positive and negative pairs, as shown in Equation (11). This formulation encourages the model to maximize mutual information between nodes that are shared between subsets (and are truly supposed to be neighbours, as these nodes share a neighbour across views) while minimizing mutual information between all other randomly paired nodes from different subsets. The rationale for this is that these neighbours are sensitive to random sampling of the nodes. 


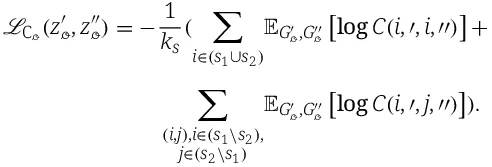
 (11)

Lastly, we use a reconstruction loss, with two examples provided in Equations (8) and (9), by concatenating (|) the input and reconstruction matrices for each of the subsets $s_{1}$ and $s_{2}$. This enables our model to learn how to rebuild information from the incomplete graphs $G^{\prime }$ and $G^{\prime \prime }$. We then compute a weighted sum of the reconstruction losses and the contrastive losses, with the reconstruction losses weighted by *α * and the contrastive losses by *β *. This formulation yields the total loss $\mathscr{L}$, as defined in Equation (12). 


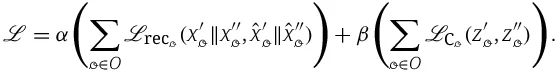
 (12)

Overall, this framework provides a scalable and efficient approach for learning joint representations from multi-omics data, even in scenarios with large sample sizes or incomplete data.

### Complexity analysis for subset contrastive learning

We analyse the time and space complexity of the proposed subset contrastive learning framework to demonstrate its efficiency over full-batch network training. Modern graph-based deep learning frameworks typically rely on edge-centric message-passing operations for graph neural networks. In our analysis, we consider a graph *G=(V,E)*, where *n=|V|*, *m=|E|* and where *d* is the number of features in our network.

Theorem 1. For a sampling rate $k_{s} \leq \frac{n}{2}$ and linear or superlinear operations, the memory required to store the values is at most a constant factor greater than that required for the full graph with *n* nodes.

Proof. Given $k_{s} = \frac{n}{2}$, we have two graphs, $G^{\prime } = (V^{\prime },E^{\prime })$ and $G^{\prime \prime } = (V^{\prime \prime },E^{\prime \prime })$, each of size $k_{s}$. Since $G^{\prime }$ and $G^{\prime \prime }$ are equally sized, we can assume, without loss of generality, that all operations take double the time and space required by $G^{\prime }$. By definition, we have $|V^{\prime }|=k_{s}=\frac{n}{2}$ nodes in our subsampled graphs. Using KNN graphs with $k_{k}$ edges per node, the number of edges in the subsampled graphs is $|E^{\prime }|\approx k_{s}k_{k}=\frac{k_{k}n}{2}$. Therefore, for any operation *f* takes linear time/space w.r.t. *n* (*O(f)=O(n+c)*) or *m* (*O(f)=O(m+c)*), the time required for both subgraphs is:

(13) \begin{align*}& O(f^{\prime}) = O\left(2\times \left(\frac{n}{2}+c\right)\right) = O(n+2\times c).\end{align*}

equivalently for operations depending on the number of edges. Therefore, *O(f)* and $O(f^{\prime })$ are equal up to a constant factor. For operations with superlinear complexity ($k_{p}>1$), the time required becomes:

(14) \begin{align*}& O(f^{\prime}) = O\left(2\times \left(\left({\frac{n}{2}}\right)^{k_{p}}+c\right)\right) = O( {n}^{k_{p}} \times{2}^{1-k_{p}} + 2\times c)).\end{align*}

Since $k_{p}>1$, $1-k_{p}<0$, meaning that the multiplicative factor ${2}^{1-k_{p}}$ reduces overall growth, while the constant factor increases by at most 2. A similar argument can be followed for other cases.

Many contrastive learning methods require either a linear number of comparisons (e.g. DGI [28]) or a quadratic number of comparisons, where each node is contrasted with every other node (e.g. [15, 24, 29]). Our analysis demonstrates that, under a sampling size smaller than $\frac{n}{2}$, our method requires less memory for these linear-to-superlinear contrastive learning strategies. Although the reduction factor depends on the ratio $\frac{k_{s}}{n}$, the improvement in memory usage is particularly crucial for datasets with large sample sizes, where the existing, full-graph algorithms become computationally prohibitive. For instance, the implementation of the SURER algorithm [15] exceeded available memory resource (*≥ 64* GB) when applied to the dataset from [35], whereas SCONE completed training without issue.

These empirical observations are consistent with the theoretical results presented in Theorem 1, which show that the subset-contrastive formulation scales at most linearly with the number of samples and achieves sublinear memory growth relative to conventional full-GCL, which is highlighted in Fig. S9. We also provide results for the case where someone has a fixed memory budget, for an increasingly large dataset in Fig. S10, and for increasing subset sizes with a fixed dataset size in Fig. S11.

From a computational perspective, models such as TotalVI [25] operate directly on feature matrices using VAE architectures and, therefore, do not require graph construction or message-passing operations, typically resulting in lower per-epoch computational overhead. By contrast, SCONE introduces additional computation through graph-based encoders and subset-based contrastive learning. However, the subset-sampling strategy enables scalable graph-based representation learning while keeping memory usage and runtime practical for large datasets. Therefore, one of the main contributions of SCONE is the subset-contrastive objective which enables subset-sampled training to be used with graph-based methods, thereby facilitating scalable graph-based learning on large datasets.

## Results and discussion

We evaluated the SCONE model across both single-cell and bulk multi-omics datasets to assess its ability to integrate heterogeneous molecular modalities, preserve biological structure, and scale efficiently. The analyses below examine SCONE performance on the Kotliarov2020 single-cell dataset and multiple TCGA cancer cohorts, followed by an assessment of latent structure, cross-omic behaviour, and integration synergy.

### Single-cell multi-omics integration

Table 3 highlights the strong performance of SCONE, in comparison to the baseline TotalVI on the Kotliarov2020 dataset [35], whereas methods such as SURER [15] fail to run due to their quadratic memory requirements. SCONE achieved the highest average adjusted mutual information (AMI) and adjusted rand index (ARI) values when integrating both RNA and CITE-seq omics layers with the Leiden clustering algorithm. Although differences were not statistically significant compared with TotalVI under the same conditions, SCONE demonstrated competitive performance within each clustering algorithm. Notably, SCONE RNA+CITE excelled in nearly all scenarios, with the exception of AMI under the Louvain algorithm, where TotalVI using only CITE-seq slightly outperformed.

**Table 3:** AMI and ARI in the Kotliarov2020 dataset [35].

Model	Views	Louvain		Leiden	
		AMI	ARI	AMI	ARI
KOTLIAROV2020 [35]					
TotalVI [25]	RNA	*0.692± 0.015*	*0.561± 0.029*	*0.703± 0.015*	*0.570± 0.030*
TotalVI [25]	CITE	$\mathbf{0.781}\pm \mathbf{0.007}$	$\textit{0.630}\pm \textit{0.022}$	$^{\mathrm{a}}\textit{0.795}\pm \textit{0.017}$	*0.651± 0.045*
TotalVI [25]	RNA+CITE	$^{\mathrm{a}}\textit{0.746}\pm \textit{0.160}$	$^{\mathrm{a}}\textit{0.618}\pm \textit{0.208}$	$^{\mathrm{a}}\textit{0.756}\pm \textit{0.163}$	$^{\mathrm{a}}\textit{0.639}\pm \textit{0.214}$
SURER	RNA+CITE	OOM	OOM	OOM	OOM
SCONE (ours)	RNA	*0.514± 0.033*	*0.361± 0.048*	*0.567± 0.040*	*0.458± 0.052*
SCONE (ours)	CITE	*0.754± 0.018*	*0.480± 0.056*	*0.777± 0.008*	*0.539± 0.023*
SCONE (ours)	RNA+CITE	*0.765± 0.017*	$\mathbf{0.654}\pm \mathbf{0.038}$	$\mathbf{^{\mathrm{b}}0.800}\pm \mathbf{0.014}$	$^{\mathrm{b}}\mathbf{0.698}\pm \mathbf{0.015}$

Values are reported as $\mathrm{mean}\pm \mathrm{SD}$ with highest mean for each metric and clustering algorithm in bold and italicized values represent results that were not statistically different (*<0.05*) from the highest value on a two-tailed t-test, either due to a tie or lack of samples. For each metric, $^{\mathrm{b}}$ and $^{\mathrm{a}}$ denote the best and non-significantly different values, respectively. SCONE used *k*=15, which lies within a stable performance region (Fig. S8). OOM denotes that a model could not be trained within the available memory resources (64GB RAM).

Interestingly, TotalVI exhibited higher average AMI and ARI values when using only CITE-seq data, than when using RNA or both omics layers combined. In contrast, SCONE consistently achieved the best performance when integrating both RNA and CITE-seq, followed by CITE-seq alone, and lastly, RNA. As expected, the quadratic memory requirements of SURER made it infeasible to train under our 64 GB memory constraint.

### Bulk cancer subtyping across The Cancer Genome Atlas multi-omics datasets

Table 4 summarizes the performance of SCONE and Subtype-MGTP across seven TCGA cancer datasets, where both methods outperformed the SURER method. SCONE demonstrated strong versatility and effectiveness, achieving the highest overall mean $-\log _{10}(P)$ values across datasets. The SCONE multi-omics configuration achieved the highest average $-\log _{10}(P)$ values in four of the seven datasets, including a statistically significant improvement over Subtype-MGTP in the SKCM dataset (*P<.05*). Both models specifically designed for omics integration outperformed the generalist SURER method, which was competitive only on the SKCM dataset.

**Table 4:** Survival log-rank test $-\log _{10}(P)$ values for the TCGA dataset [24] using *k* clusters as per [20, 24].

model	BLCA	BRCA	KIRC	LUAD	SKCM	STAD	UCEC
Subtype-MGTP	$^{\mathrm{a}}\mathbf{2.04}\pm 0.57$	$\textit{0.94}\pm 0.44$	$\textit{4.76}\pm 2.95$	$\mathbf{0.98}\pm 0.34$	*2.59± 0.72*	$^{\mathrm{a}}\mathbf{2.75}\pm 1.20$	$^{\mathrm{b}}\textit{5.14}\pm 1.52$
SURER	*1.13± 0.86*	*0.36± 0.34*	*1.30± 1.04*	*0.57± 0.70*	*2.12± 1.31*	*0.70± 0.75*	*1.17± 1.13*
SCONE (ours)	$^{\mathrm{b}}\textit{1.92}\pm 0.30$	$\mathbf{1.18}\pm 0.38$	$\mathbf{6.12}\pm 1.60$	$\textit{0.77}\pm 0.62$	$^{\mathrm{b}}\mathbf{4.69}\pm 0.78$	$^{\mathrm{b}}\textit{2.40}\pm 0.97$	$^{\mathrm{b}}\mathbf{5.92}\pm 0.65$
SCONE (rna)	$^{\mathrm{b}}1.66\pm 0.40$	$^{\mathrm{a}}1.81\pm 0.45$	$^{\mathrm{a}}10.16\pm 2.25$	$^{\mathrm{a}}2.95\pm 1.23$	$^{\mathrm{a}}4.85\pm 1.18$	*0.61± 0.45*	*5.22± 0.99*
SCONE (prot)	*1.17± 0.61*	*1.14± 0.64*	*7.46± 1.41*	*0.19± 0.18*	*2.20± 0.52*	*1.65± 0.33*	*4.33± 0.85*
SCONE (miRNA)	$^{\mathrm{b}}1.84\pm 0.51$	*0.52± 0.30*	*4.43± 2.47*	*0.64± 0.42*	*3.32± 0.55*	*0.75± 0.45*	*2.06± 0.72*
SCONE (meth)	*0.66± 0.20*	*0.70± 0.25*	*3.83± 1.32*	*1.39± 1.12*	*0.41± 0.29*	*0.09± 0.09*	$^{\mathrm{a}}6.47\pm 1.14$
SCONE (CN)	*0.08± 0.07*	*0.15± 0.07*	*2.83± 0.92*	*0.56± 0.44*	*0.46± 0.20*	*0.45± 0.39*	*1.08± 1.47*

Values are reported as $\mathrm{mean}\pm \mathrm{SD}$ (see Table S1 for $\mathrm{mean}\pm \text{standard deviation} (\mathrm{max})$). For each dataset, the highest value in the multi-omics setting is shown in bold. Italicized values indicate results that were not statistically significantly different (*<0.05*) from the highest value on a two-tailed t-test. Results obtained by SCONE are presented both for multi-omics (using RNA, Protein and miRNA), as well as for single-omics, while we follow description for Subtype-MGTP [24]. Single-omics results for SURER can be found in Table S1. Symbols $^{\mathrm{a}}$ and $^{\mathrm{b}}$ denote the best result and values non-significantly different, respectively, when considering both single- and multi-omics analyses

SCONE’s single-omics models also performed remarkably, outperforming both its multi-omics counterpart and Subtype-MGTP in five datasets based on $-\log _{10}(P)$ values. In BRCA, KIRC, and LUAD, the RNA-only SCONE model achieved significantly better results than all other models (*P<.05*). For SKCM, SCONE RNA-only outperformed Subtype-MGTP and was comparable to its multi-omics version. In UCEC, SCONE using only the methylation layer performed on par with both its multi-omics model and Subtype-MGTP, highlighting its robustness even in single-omics scenarios.

Although Subtype-MGTP achieved the highest average $-\log _{10}(P)$ in BLCA, the differences were not statistically significant when compared to SCONE’s multi-omics or single-omics models using RNA or miRNA. In STAD, Subtype-MGTP significantly outperformed SCONE single-omics models (*P<.05*) but did not achieve a significant improvement over SCONE multi-omics model, further underscoring SCONE’s competitive performance and adaptability across diverse molecular profiles.

These results suggest that the benefit of multi-omics integration depends on whether different molecular layers provide complementary prognostic information, whereas datasets dominated by a single informative modality may not benefit substantially from integration. Together with the single-cell experiments in Section Single-cell multi-omics integration, these findings demonstrate that SCONE generalizes effectively across distinct cancer types and omics modalities. We next provide further analysis of the integration behaviour and consistency across datasets.

### Consistency and integration behaviour across datasets

We now analyse the results presented in Sections Single-cell multi-omics integration and Bulk cancer subtyping across The Cancer Genome Atlas multi-omics datasets to identify consistent patterns in model behaviour across datasets. SCONE demonstrated robust performance across multiple datasets, and it was able to integrate multi-omics data when complementary signal was present, while maintaining stable performance in single-omics-dominant settings. On the Kotliarov2020 dataset, SCONE exhibited a narrower performance distribution compared with TotalVI multi-omics model, indicating a more consistent clustering performance. Notably, TotalVI achieved its best performance using only the CITE-seq data, which were also used to define the dataset’s “ground truth” labels [35]. As a result, clustering metrics may favour models trained on the protein modality used to derive the reference labels. In contrast, SCONE multi-omics integration strategy enabled a synergistic interaction between omics layers, effectively leveraging complementary information to outperform models utilizing only a single omics layer. For example, SCONE integration of RNA and CITE-seq data compensated for potential noise in individual layers, allowing it to achieve superior performance compared with models relying solely on the CITE-seq layer.

### Latent space structure and biological interpretability

The ability of SCONE to capture meaningful biological insights is reflected in the structure of its learned latent manifold. As illustrated in Fig. 2, the joint RNA+CITE-seq model produced well-defined clusters corresponding to biologically coherent immune cell populations. These clusters demonstrated clear separation at both broad and fine cell-type annotation levels, consistent with the reference cell-type definitions reported by Kotliarov *et al*. [35].

**Figure 2.**
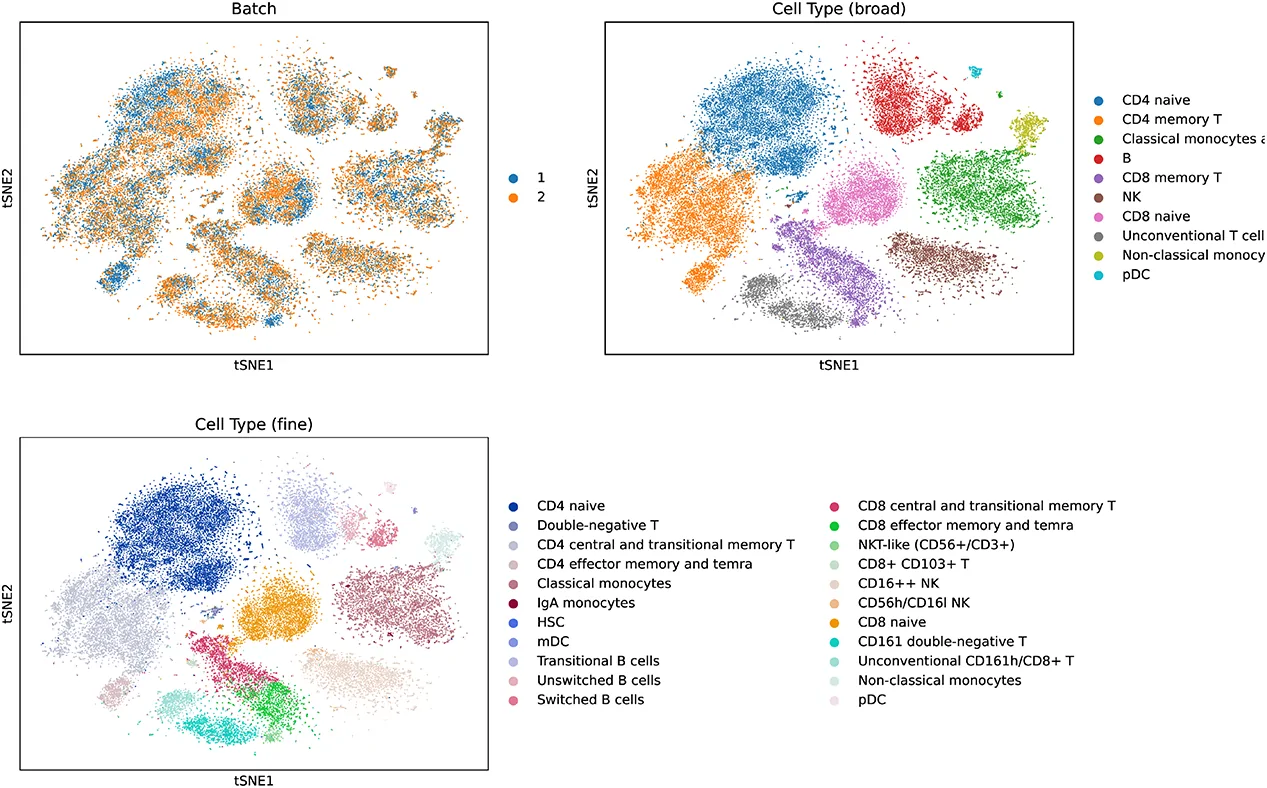
t-SNE [46] visualization of the latent representation learned by the SCONE multi-omics model on the Kotliarov2020 dataset [35]. Clockwise from top left: **Batch**: batch assignments defined by Lotfollahi *et al*. [26]; Cell Type (broad), broad cell-type annotation; and **Cell Type (fine)**, fine cell-type annotation. Both broad and fine cell type annotations were defined computationally by Kotliarov *et al*. [35]. Representation generated by SCONE clearly distinguishes both broad and fine cell types while maintaining alignment across batches, revealing distinct substructure within major immune cell populations.

Although single-omics SCONE models underperformed slightly compared with TotalVI’s single-omics models, the multi-omics integration achieved superior overall biological resolution. This suggests that SCONE’s subgraph-based contrastive learning effectively leverages complementary information between omic layers to refine cell-type boundaries and reduce the influence of noise or sparsity within individual modalities. We hypothesize that the subset sampling procedure introduced minor stochastic variation in single-omics embeddings, which may explain the modest performance differences. Nevertheless, SCONE’s multi-omics integration compensates for such effects by aligning shared signal across layers, resulting in a more stable and biologically interpretable representation.

This observation is further supported by the visualization of surface-protein cell-type markers [35] in Fig. 3 that show clear expression patterns within each cell type cluster. Notably, even HSC markers, representing only 57 cells (0.11% of the dataset), formed a distinct and compact cluster in the centre of the t-SNE visualization [46], underscoring the ability of our model to preserve biological information in its learned manifold, even for rare cell populations. Together, these findings confirm that SCONE’s latent manifold captures meaningful biological variation while maintaining coherence across diverse and imbalanced immune cell populations.

**Figure 3.**
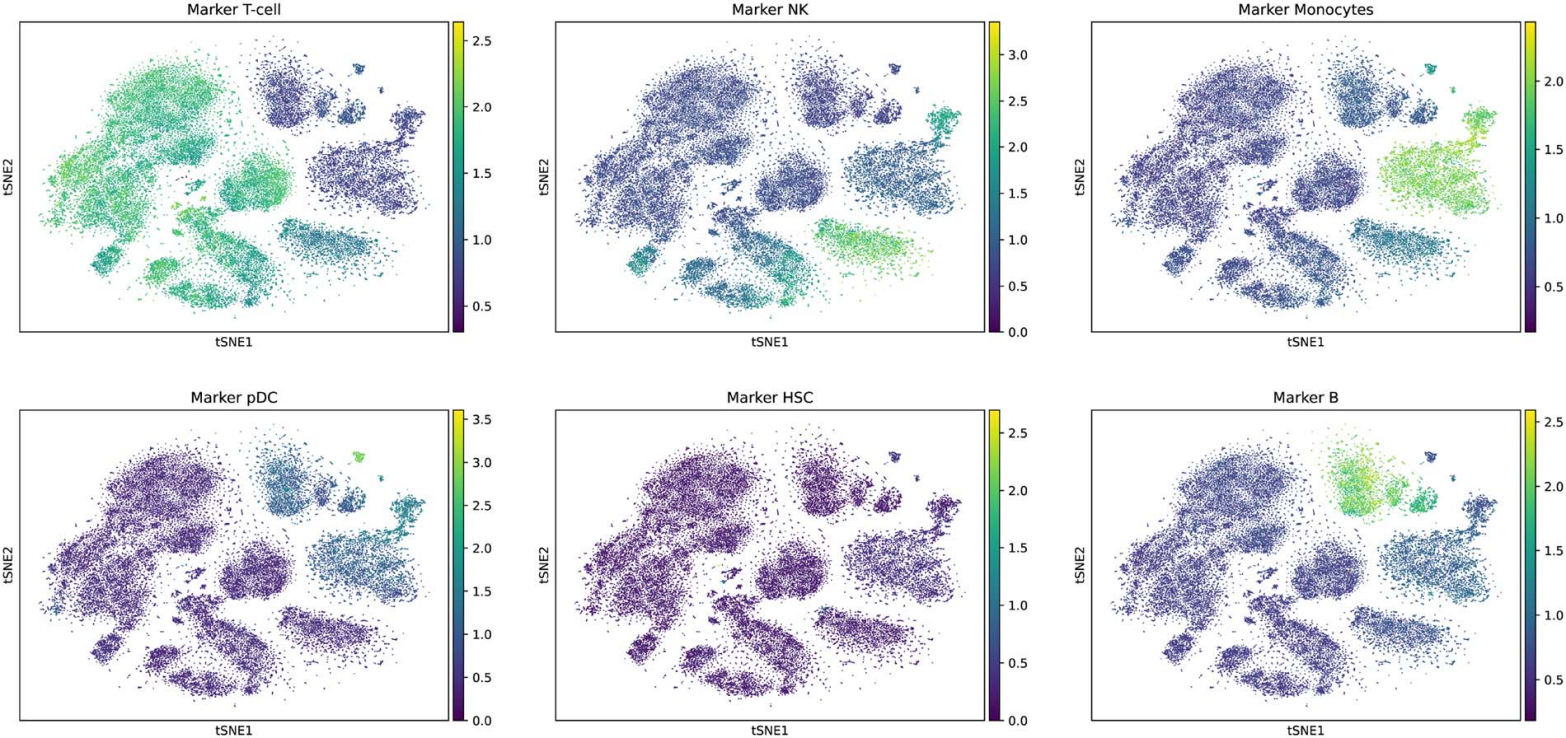
Expression patterns of selected surface protein markers projected onto the SCONE latent space for the Kotliarov2020 PBMC dataset. Each panel shows marker intensity across the embedding, illustrating the correspondence between protein expression and inferred cell-type clusters, Panels, from left to right and top to bottom, represent cell markers for: Marker T-cell, T-cells; Marker NK, Natural Killer cells; Marker Monocytes, Monocytes; Marker pDC, Plasmacytoid Dendritic Cells; Marker HSC, hematopoietic stem cells; Marker B, B cells. Strong, localized expression of canonical markers for T-cells, Monocytes, Natural Killer (NK) cells, plasmacytoid dendritic cells (pDC), HSC, and B-cells, confirms that SCONE preserves biologically interpretable relationships in its learned manifold, even for rare populations such as hematopoietic stem cells (see Section S2 for further discussion). Additional marker–protein visualizations demonstrating that the integrated latent topology preserves key surface protein information are provided in Supplementary Materials (Figs S1–S6).

### Cross-omic behaviour and dataset-specific patterns

Across TCGA datasets, SCONE exhibited consistent and biologically interpretable cross-omic behaviour. Single-omics RNA models consistently yielded higher $-\log _{10}(P)$ values compared with protein and generalist models, indicating that transcriptomic features captured much of the relevant variation for patient survival. The main exception was the STAD dataset, where protein markers were particularly informative for patient survival and protein-based models achieved highest performance. This pattern aligns with Subtype-MGTP’s multi-omics-to-protein translation framework, which assumes that the protein omics layer provides the most informative signal [24].

Our experiments suggest that this assumption holds primarily in contexts where protein-level regulation is strongly associated with clinical outcomes, such as in STAD. In that dataset, both SCONE’s protein-only model and Subtype-MGTP achieved superior $-\log _{10}(P)$ values relative to RNA- or methylation-based models, indicating a prominent role for protein-level signalling in gastric tumour biology. In contrast, in several other cancer types (BRCA, KIRC, and LUAD), SCONE’s RNA-only models achieved higher $-\log _{10}(P)$ values, suggesting that transcriptomic signals were more predictive of disease subtypes and outcomes in these settings. Notably, UCEC exhibited a distinct pattern, with methylation-based models achieving performance comparable to the best-performing configurations, highlighting the context-dependent relevance of individual omics layers.

In terms of contextualizing the observed performance differences across cancer types, a noteworthy dataset-specific pattern is observed in KIRC, where the RNA-only SCONE model substantially outperforms the multi-omics configuration (Table 4). This behaviour is biologically well grounded, as KIRC is molecularly defined in most cases by biallelic inactivation of the VHL tumour suppressor gene [47], leading to constitutive activation of hypoxia-inducible factor transcriptional programmes [48]. These programmes generate a highly coherent transcriptomic signal governing hypoxia response, angiogenesis and metabolic reprogramming, which has been shown to be strongly predictive of clinical outcome [49]. Consequently, integrating additional omics layers may dilute this dominant prognostic axis rather than enhance it.

A complementary dataset-specific pattern is observed in UCEC, where the methylation-only SCONE model achieves performance comparable to the best-performing configurations (Table 4). This result is consistent with the established biology of endometrial cancer, which is characterized by pronounced epigenetic heterogeneity. DNA methylation plays a central role in defining key molecular subgroups in this disease, including hypermethylated phenotypes linked to microsatellite instability, as described by TCGA [50], and has also been identified as an independent prognostic factor in endometrial cancer [51]. As a result, methylation profiles capture prognostically relevant structure that may be less prominent at the transcriptomic or proteomic levels.

A further context-specific pattern is observed in STAD, where protein-based models achieve superior performance relative to RNA- and methylation-based approaches (Table 4). This observation aligns with the molecular biology of gastric cancer, which is characterized by substantial heterogeneity driven by signalling pathway dysregulation and post-transcriptional regulatory mechanisms, leading to distinct clinical outcomes across molecular subtypes [52]. In particular, aberrant activation of receptor tyrosine kinases and downstream signalling cascades is often more directly reflected at the protein, including phosphoprotein, level than in mRNA abundance alone, as demonstrated by recent proteogenomic analyses of gastric adenocarcinoma [53]. Accordingly, proteomic data capture clinically relevant variation that is particularly informative for survival stratification in this cancer type.

Taken together, the RNA-dominant behaviour observed in KIRC, the methylation-driven stratification in UCEC, and the protein-informative patterns in STAD illustrate that the optimal molecular modality for survival prediction is intrinsically linked to the dominant pathogenic mechanisms of each cancer type. These results highlight that no single omics layer is universally optimal; rather, predictive signal strength is context-dependent and shaped by disease-specific biology.

Importantly, SCONE maintained strong performance in both single-omics and integrated configurations, demonstrating that its subgraph-based learning strategy adapts to dataset-specific modality relevance rather than overfitting to a single omic layer. This flexibility enables SCONE to generalize effectively across diverse biological and clinical contexts, providing interpretable latent representations that reflect the dominant molecular mechanisms within each dataset.

### Synergy and robustness of multi-omics integration

While multi-omics integration in SCONE did not always produce synergistic improvement, it often provided an advantage over analysing omics layers individually or averaging single-omics results. Specifically, we consider that a model provided synergistic omics integration if its downstream performance is better than if we trained individual single-omics models, and average their prediction: a single-omics ensemble. This differs from cases where an improvement over any single-omics layer is always required, but we believe that our definition is useful for cases such as unsupervised multi-omics integration, where the predictive endpoint is unknown and different omics layers may be more informative against different predictive endpoints.

Synergistic behaviour was most evident in three datasets, namely BLCA, STAD, and UCEC, where the integrated model achieved higher average performance than any single-omics variant. This pattern suggests that integration is most beneficial when distinct molecular modalities capture complementary aspects of tumour biology. In three other datasets, the average performance of single-omics models (RNA, Protein, and miRNA) was within 1 SD of the multi-omics model, suggesting that SCONE maintains stable integration even when synergy is modest.

Specifically, in the BRCA dataset the single-omics ensemble was *0.05σ * below the multi-omics model, while in the KIRC and LUAD datasets, the single-omics ensemble outperformed the multi-omics model by *0.77σ * and *0.79σ *, respectively. These differences are consistent with dataset-specific modality dominance discussed earlier (Section Cross-omic behaviour and dataset-specific patterns). For example, transcriptomic programmes dominate prognostic variation in KIRC, whereas methylation-driven stratification in UCEC and protein-level signalling variation in STAD provide complementary information across modalities. Conversely, in SKCM, despite the RNA-seq-only model achieving the highest average $-\log _{10}(P)$ value, the single-omics ensemble performed *1.58σ * below the multi-omics model.

Taken together, these results indicate that the benefit of multi-omics integration depends on the relative strength, complementarity and noise characteristics of modality-specific signals. When multiple omics layers capture distinct but related biological processes, integration improves representation learning, while in cases when a single modality dominates the prognostic signal, single-omics models may perform comparably. Importantly, SCONE’s subset-contrastive learning framework allows the model to adapt to these conditions without enforcing integration when additional modalities provide limited complementary information.

In summary, SCONE achieved competitive or superior performance to existing state-of-the-art models while maintaining near-linear scalability and reduced memory consumption. Its subset-contrastive design enables efficient learning from large, heterogeneous datasets without sacrificing biological coherence. By jointly embedding heterogeneous omic layers, SCONE captures subtle cross-omic relationships while preserving dominant modality-specific structure when integration provides limited benefit. These properties establish its potential as a powerful analytical framework for integrative multi-omics and precision medicine applications.

## Conclusions

We introduce SCONE, a scalable framework for multi-omics data integration that demonstrated robust and consistent performance across both single-cell and bulk datasets. By employing a subgraph-based contrastive learning strategy, SCONE effectively integrates multiple omic layers, capturing complementary biological signals while maintaining computational efficiency. Despite the stochasticity introduced by subset sampling, the model consistently matched or exceeded the performance of existing network-based and nonnetwork-based approaches.

Our findings highlight the promise of subgraph-contrastive learning for large-scale molecular data. SCONE integrates heterogeneous omics modalities efficiently with reasonable computational overhead while enabling scalable graph-based representation learning, features that are particularly important in multi-omics research. Results underscore the potential of subset-based methods for challenges inherent in emerging data modalities, such as spatial transcriptomics and single-cell multi-omics, where data sparsity and resolution constraints are particularly pronounced.

While SCONE demonstrates strong performance across diverse datasets, several practical considerations should be noted. Performance depends on graph construction choices and on the presence of complementary signal across omics modalities. Graph-based encoders also introduce modest computational overhead relative to matrix-based autoencoder approaches. SCONE provides a practical trade-off between computational cost and modelling flexibility, enabling scalable graph-based multi-omics integration for datasets where full-graph contrastive methods may become computationally prohibitive. Our results further indicate that the benefits of multi-omics integration are dataset dependent, while the contrastive objective primarily acts as a representation-alignment and regularization mechanism within the SCONE framework.

These considerations motivate several directions for future work. We anticipate that SCONE will motivate further research into advancing contrastive learning in systems biology, and enable efficient, biologically grounded representation learning. Future work may focus on refining subset selection and integration strategies to enhance cross-omic synergy, as well as extending SCONE’s framework to accommodate the specific demands of spatially resolved and temporally dynamic datasets. Together, these directions have the potential to expand the applicability of SCONE to new frontiers in precision medicine and data-driven biological discovery.

**Key points**

- We propose SCONE, a scalable framework that adapts contrastive learning to multi-omics data integration using a subgraph-based approach.
- SCONE effectively converts the inherent pairwise similarity basis of omics graphs into a strength, enabling robust representation learning with near-linear scalability and reduced memory consumption compared with full-graph methods.
- Extensive experiments show our method is applicable in both single-cell and bulk multi-omics settings, and can capture meaningful representations even in rare cell populations.
- In clustering on a single-cell dataset, SCONE’s multi-omics model demonstrated a synergistic interaction between omics layers that often outperformed models using single layers alone.
- SCONE’s bulk omics integration achieved the highest overall mean $-\log _{10} P$-values across all tested omics configurations in four of the seven datasets.

## Supplementary Material

BIB-25-2248_R2-suppl_bbag381

## Data Availability

Datasets used in this study are publicly available. The Protein information for the TCGA dataset used is available at github.com/kybinn/Subtype-MGTP, with an archived version hosted at github.com/phcavelar/Subtype-MGTP, all other omics layers are available at https://github.com/haiyang1986/Subtype-GAN, with an archived version hosted at https://github.com/phcavelar/Subtype-GAN. The Kotliarov2020 dataset is available at https://drive.google.com/drive/folders/1CTYgH42BY71HUjnG_K7MYpO6VJfaAAlK?usp=sharing. Source code is available at github.com/phcavelar/network-multi-omics (archived at github.com/KISysBio/network-multi-omics).
