# Supplementary material for: SCONE: a subset-contrastive method for multi-omics network embedding
Source: Brief Bioinform. 2026 Aug 12;27(4):bbag381. doi: 10.1093/bib/bbag381 (PMC13631930; doi:10.1093/bib/bbag381)
Supplement: Supplementary file 1 [file BIB-25-2248_R2-suppl_bbag381.pdf]

# Supplementary Material for "SCONE: Subset-Contrastive Multi-Omics Network Embedding"

## Experimental Setup

We selected three strong baselines for comparison, with two specifically built for multi-omics data: (i) TotalVI [25] and (ii) Subtype-MGTP [24]. TotalVI is a widely recognised multi-omics single-cell representation learning method, extensively used and integrated into several established and emerging libraries [54, 55, 56]. Subtype-MGTP, on the other hand, is a recently published multi-omics bulk representation learning method that has demonstrated significant improvements over prior techniques. We also compare with a third baseline (iii), a generalist multi-view (i.e. multi-omics) baseline, SURER [15] which has shown state-of-the-art performance at a top AI conference. During the development of our work, SURER's authors also published a different model called SERIES [16], but we were unable to use it due to its source code not being made available. For all baselines, we used the authors' publicly available code and adhered to their recommended settings. All training was done using a NVIDIA RTX A4000 GPU with 8 GB of VRAM, if a model failed to fit inside the GPU, we further attempted training it on a CPU with 64GB of memory.

### *Baseline Models*

TotalVI is a multi-modal Variational Autoencoder [57] tailored for RNAseq and CITE-seq (surface protein) data. It employs a standard normal prior and reconstructs RNAseq data using a Negative Binomial (NB) distribution, while protein data are reconstructed using an NB mixture. We utilised the implementation available in the `scvi` Python package [54] (version `0.14.6`), produced by the same first author as the original TotalVI paper. The latent representation was extracted using the `get_latent_representation()` method of the `TOTALVI` class.

Subtype-MGTP is a two-phase model. The first phase trains a multi-omics-to-protein translation module using Graph Convolutional Network (GCN) [58] layers on all available protein data. Then it converts all multi-omics measurements into protein representations, which are then used as input for the second phase. In the second phase, Subtype-MGTP builds a Autoencoder model comprising GCN-based Encoder and Decoder, and then applies a Deep Subspace Contrastive Clustering module, along with a contrastive self-expression layer in the latent space. We used the original implementation available at `github.com/kybinn/Subtype-MGTP`, with an archived version hosted at `github.com/phcavelar/Subtype-MGTP`.

SURER is a generalist Multi-view (here, multi-omics) graph contrastive Learning method [15] which works by iteratively refining each view's affinity (adjacency) matrix. It first uses view-specific graph autoencoders that compresses the features of each layer to reconstruct each individual view graph. Then it builds a heterogeneous graph by linking all view-specific graphs, which is then input in a heterogeneous graph neural network to produce a consensus representation. Finally, the consensus representation is used for clustering. However, due to the optimisation objective including the reconstruction of the adjacency matrix, SURER is limited by its quadratic memory requirement, which prevented it from being applied on our single-cell dataset. We used the original implementation at `github.com/Wjing-bjtu/SURER`, with an archived version hosted at `github.com/phcavelar/SURER`.

### *Proposed Model*

Our model employs mirrored encoders and decoders, with each omics layer's encoder consisting of two GATv2 layers with 256 and 128 units, followed by LeakyReLU activation. The decoder mirrors this structure, with 256 units in the first layer and $d_o$ output units. Training was performed using the Adam optimiser [59] with $\beta_1 = 0.9$, $\beta_2 = 0.999$, and a learning rate of 0.0001. We used $\alpha = 1$ and $\beta = 10$ in Equation 12. We did not optimise our model's hyperparameters for each experiment, only using different values for $k_s$ and number of epochs, as detailed below. For subset sampling, we ensured that at least 10% of nodes overlapped between subsets ($|s_1 \cap s_2| \geq 0.1 \times k_s$).

Post-training, we performed clustering using either Louvain [60] or Leiden [61] methods. For Louvain clustering, we used the `louvain_communities` function from the `networkx` library [62] (version `3.3`). For Leiden clustering, we used the `find_partition` function from the `leidenalg` library [61] (version `0.9.1`). The resolution parameter was varied over 20 values in the $(0, 2]$ interval, selecting the clustering with the highest modularity. KNN graphs (k=15) were constructed where choice of k was in line with common practice in graph-based single-cell and multi-omics modelling [63] and further validated through sensitivity analysis (Figure S8) using the `nn.neighbors` function from the `scanpy` library [43] (version `1.10.2`). Increasing the neighbourhood parameter $k$ beyond this value led to reduced AMI and ARI performance, likely due to denser neighbourhood graphs and thereby decreasing clustering separability.

### *Single-Cell Data*

For the single-cell dataset [35], we trained each model using RNA and CITE-seq data individually and combined (RNA+CITE). TotalVI was adapted to single-omics scenarios by providing a dummy matrix of zeros for the missing omics layer, with a single column containing zeros for all samples. We used subsets containing 20% of the dataset ($k_s = 0.2 \times n$), corresponding approximately to the proportion of the most abundant cell type, and trained the models for 128 epochs. Both Louvain and Leiden clustering were applied, and results were reported separately.

Cluster assignments were evaluated using the Adjusted Rand Index (ARI, Equation 15) [64] and Adjusted Mutual Information (AMI, Equation 16) [65], calculated using the `adjusted_rand_score` and `adjusted_mutual_info_score` functions from the `scikit-learn` library [66] (version `1.4.2`). The cluster assignments ($y_{pred}$) were compared with the original clustering labels ($y_{true}$) provided by [35] for ARI and AMI calculation, which were used as the performance indicator for each model. Cluster assignments were compared to the original labels provided by [35]. Each model was trained eight times, and clustering was performed on each latent space, yielding eight clusterings per model/clustering method combination. We report the mean and standard deviation of ARI and AMI for each configuration.

$$ARI(y_{true}, y_{pred}) = \frac{2(TP \cdot TN - FN \cdot FP)}{(TP + FN)(FN + TN) + (TP + FP)(FP + TN)} \tag{15}$$

Where $TP$, $TN$, $FP$, and $FN$ are the counts of true positive, true negative, false positive, and false negative pairs, respectively, as determined by the pair confusion matrix.

$$AMI(y_{true}, y_{pred}) = \frac{MI(y_{true}, y_{pred}) - E[MI(y_{true}, y_{pred})]}{\frac{1}{2}(H(y_{true}) + H(y_{pred})) - E[MI(y_{true}, y_{pred})]} \tag{16}$$

Where $MI$ is the Mutual Information, $H$ is the Shannon Entropy, and $E[MI]$ is the Expected Mutual Information between two random clusterings.

### *Batch Data*

For Batch data, we followed the preprocessing steps in [24]. Model performance was assessed using the logrank p-value of survival separation, calculated with the `multivariate_logrank_test` function from the `lifelines` library [67] (version `0.30.0`). A pre-defined number of clusters for each cancer subtype was used, as defined by [20] and shown in Table 2, for fair comparison. Unlike [24], we trained models separately for each cancer subtype, ensuring no information flowed between datasets, and conducted multiple tests for each model. We chose to use a fixed value of $k_s = 200$ samples per subset for each of the datasets, as this allowed us to have a consistent number of samples and $k_s = 200$ is close to half the average dataset size (datasets had on average 215 samples). We trained each model for 1,024 epochs, as smaller sample sizes allowed for faster training compared to the single-cell case.

As with single-cell data, we ran each model 8 times, extracted its latent space, and performed clustering on the latent space. To keep consistent with our single-cell experiments, we only used the equivalent omics layers encoding RNA, Protein, and Micro-RNA when training the model in a multi-omics scenario, also providing the performance for each omics layer when used individually. Since the Leiden clustering algorithm provides an improvement over Louvain [61] and is faster to compute, we decided to only perform clustering with Leiden. Since Leiden clustering does not provide a clustering with a fixed number of clusters, we adapted our optimisation procedure and only considered clusterings that had the target number of clusters. After clustering, we calculated the logrank p-value of the clusters' survival separation. For Subtype-MGTP, we follow its second phase and used spectral clustering with the pre-specified number of clusters for their model. We report the mean and standard deviation of the negative $\log_{10}$ p-value for fair comparison. We also include the highest values we achieved for each of the models since, given our results using the code provided by [24], we believe that they reported their best model instead of an average value over multiple runs.

## On rare cell populations

We highlight SCONE's ability to preserve rare cell populations despite the model never accessing the whole dataset in a single backward pass. We consider a cell type as "rare" if it makes up less than 1% of the total dataset size, and "common" otherwise.

In the Kotliarov2020 dataset [35], the broad cell types had an average silhouette score of 0.47, which also remained when considering only the common cell types and pDC cells had a silhouette score of 0.82. For the fine cell type classification, the average silhouette score was 0.47, with common cell types having an average score of 0.37 and rare cell types having a lower, but still positive score of 0.20. These

The full results can be seen in Tables S2 and S3. We note again that, although "HSC" cells had a negative silhouette score, its associated gene markers were spatially co-located, as was visualised in Figure 3.

We extend these results by using the dataset from [68], downloaded from the url `https://atlas.fredhutch.org/nygc/multimodal-pbmc/`, which we henceforth refer to as Hao2021. For the Hao2021 dataset, we used $k_s = 10\% \times n$ as the subset size for SCONE, due to its size: $n = 161,764$ cells, 3.1× larger than Kotliarov2020. The results of this analysis are discussed below, and can also be visualised in Figure S7.

The model achieved a silhouette score of 0.42 over the broad cell types (under which no cell type as considered rare). However, when analysing a finer annotation, the overall silhouette score was 0.20, and 0.11 and 0.21 for rare and non-rare cell types, respectively. So while rare cell types are harder to identify, most were still recovered with a positive silhouette score. Doublets had the lowest score and were the third most common category of rare cell types. Had doublets not been considered, the silhouette score for rare cell types would be 0.19 instead, much closer to the 0.21 for non-rare cells.

The full results can be seen in Tables S4 and S5. Table S4, specifically, shows that most broad cell types have high silhouette scores, with "CD8 T" and "other T" having smaller, but still positive scores. One can also see in Table S5 that, for finer cell type distinctions, those with more distinct markers, such as NK_CD56bright, pDC, cDC1, and Plasmablasts had higher silhouette scores.

## Supporting Figures

| model | BLCA | BRCA | KIRC | LUAD | SKCM | STAD | UCEC |
|---|---|---|---|---|---|---|---|
| Subtype-MGTP | $^{*}$**2.04** $\pm$ 0.57(2.71) | *0.94* $\pm$ 0.44(1.64) | *4.76* $\pm$ 2.95(9.12) | **0.98** $\pm$ 0.34(1.62) | 2.59 $\pm$ 0.72(4.06) | $^{*}$**2.75** $\pm$ 1.20(4.60) | $^{\dagger}$*5.14* $\pm$ 1.52(8.28) |
| SURER | 1.13 $\pm$ 0.86(2.48) | 0.36 $\pm$ 0.34(1.12) | 1.30 $\pm$ 1.04(3.57) | 0.57 $\pm$ 0.70(2.23) | 2.12 $\pm$ 1.31(4.05) | 0.70 $\pm$ 0.75(2.18) | 1.17 $\pm$ 1.13(3.70) |
| SCONE (ours) | $^{\dagger}$*1.92* $\pm$ 0.30(2.26) | **1.18** $\pm$ 0.38(1.59) | **6.12** $\pm$ 1.60(9.84) | *0.77* $\pm$ 0.62(2.14) | $^{\dagger}$**4.69** $\pm$ 0.78(5.75) | $^{\dagger}$*2.40* $\pm$ 0.97(3.80) | $^{\dagger}$**5.92** $\pm$ 0.65(6.98) |
| SURER (rna) | 0.73 $\pm$ 0.35 (1.34) | 1.11 $\pm$ 0.79 (2.93) | 5.02 $\pm$ 2.01 (6.68) | 0.85 $\pm$ 0.53 (1.71) | 1.15 $\pm$ 0.82 (2.60) | 0.52 $\pm$ 0.29 (1.14) | 1.88 $\pm$ 1.76 (4.72) |
| SURER (protein) | 0.53 $\pm$ 0.50 (1.69) | 0.34 $\pm$ 0.21 (0.58) | 1.67 $\pm$ 1.31 (3.80) | 0.20 $\pm$ 0.24 (0.78) | 1.29 $\pm$ 0.60 (2.11) | 0.49 $\pm$ 0.18 (0.82) | 2.39 $\pm$ 1.12 (3.65) |
| SURER (miRNA) | $^{\dagger}$1.69 $\pm$ 0.86 (2.93) | 0.15 $\pm$ 0.04 (0.22) | 1.48 $\pm$ 0.86 (2.87) | 0.26 $\pm$ 0.25 (0.73) | 0.85 $\pm$ 0.82 (2.28) | 1.19 $\pm$ 0.53 (2.08) | 0.65 $\pm$ 0.45 (1.39) |
| SURER (meth) | 0.90 $\pm$ 0.78 (2.44) | 0.25 $\pm$ 0.30 (0.94) | 1.44 $\pm$ 1.27 (4.37) | 0.36 $\pm$ 0.11 (0.47) | 0.74 $\pm$ 0.63 (1.85) | 0.59 $\pm$ 0.51 (1.43) | 4.04 $\pm$ 2.73 (7.61) |
| SURER (CN) | 0.43 $\pm$ 0.51 (1.40) | 0.27 $\pm$ 0.28 (0.81) | 1.76 $\pm$ 1.37 (3.89) | 0.34 $\pm$ 0.38 (1.09) | 0.32 $\pm$ 0.36 (0.90) | 0.53 $\pm$ 0.37 (1.27) | 0.50 $\pm$ 0.38 (1.10) |
| SCONE (rna) | $^{\dagger}$1.66 $\pm$ 0.40 (2.28) | $^{*}$1.81 $\pm$ 0.45 (2.51) | $^{*}$10.16 $\pm$ 2.25 (13.97) | $^{*}$2.95 $\pm$ 1.23 (4.53) | $^{*}$4.85 $\pm$ 1.18 (6.49) | 0.61 $\pm$ 0.45 (1.44) | 5.22 $\pm$ 0.99 (6.59) |
| SCONE (prot) | 1.17 $\pm$ 0.61 (2.54) | 1.14 $\pm$ 0.64 (2.04) | 7.46 $\pm$ 1.41 (9.31) | 0.19 $\pm$ 0.18 (0.51) | 2.20 $\pm$ 0.52 (3.02) | 1.65 $\pm$ 0.33 (2.28) | 4.33 $\pm$ 0.85 (5.22) |
| SCONE (miRNA) | $^{\dagger}$1.84 $\pm$ 0.51 (2.54) | 0.52 $\pm$ 0.30 (0.98) | 4.43 $\pm$ 2.47 (10.12) | 0.64 $\pm$ 0.42 (1.35) | 3.32 $\pm$ 0.55 (3.97) | 0.75 $\pm$ 0.45 (1.35) | 2.06 $\pm$ 0.72 (3.35) |
| SCONE (meth) | 0.66 $\pm$ 0.20 (0.86) | 0.70 $\pm$ 0.25 (0.94) | 3.83 $\pm$ 1.32 (5.64) | 1.39 $\pm$ 1.12 (3.19) | 0.41 $\pm$ 0.29 (0.84) | 0.09 $\pm$ 0.09 (0.27) | $^{*}$6.47 $\pm$ 1.14 (7.98) |
| SCONE (CN) | 0.08 $\pm$ 0.07 (0.20) | 0.15 $\pm$ 0.07 (0.26) | 2.83 $\pm$ 0.92 (4.22) | 0.56 $\pm$ 0.44 (1.41) | 0.46 $\pm$ 0.20 (0.68) | 0.45 $\pm$ 0.39 (1.11) | 1.08 $\pm$ 1.47 (4.64) |

**Table S1.** Survival logrank test $-\log_{10}(p)$ values for the TCGA dataset [24] using $k$ clusters with a predefined $k$ [20, 24]. Values are presented as mean $\pm$ standard deviation(max). The highest value for each dataset in the multi-omics setting is highlighted in **bold**. *Italicised* values indicate results that were not statistically significantly different ($< 0.05$) from the highest value on a two-tailed t-test. SCONE results are presented both for the multi-omics setting, where RNA, Protein and miRNA layers are used, and single-omics settings, while we follow [24]'s description for Subtype-MGTP. Symbols $^{*}$ and $^{\dagger}$ are used to denote best and non-significantly different values, respectively, when considering all single-omics sensitivity and multi-omics results.

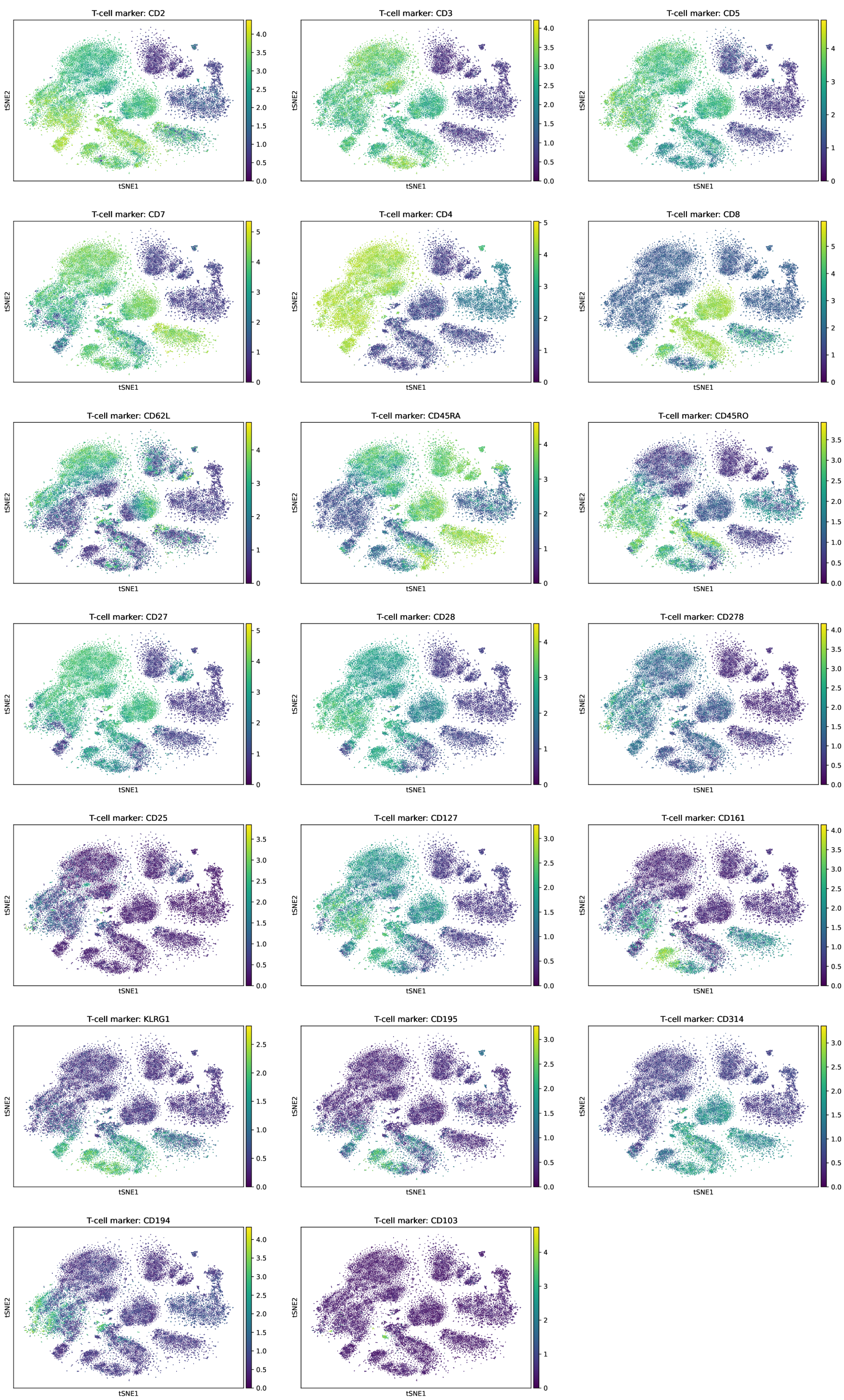


**Fig. S1.** A t-SNE [46] visualisation of one of our multi-omics model's learned latent representation of the [35] dataset. We overlay the $\log_{10}$ surface-protein expression for the marker proteins of T-cells defined by [35].

| Cell Type (broad) | Proportion (%) | Silhouette Score |
|---|---|---|
| CD4 naive | 21.35 | 0.59 |
| CD4 memory T | 17.58 | 0.21 |
| Classical monocytes and mDC | 12.72 | 0.61 |
| B | 11.15 | 0.83 |
| CD8 memory T | 10.11 | -0.11 |
| CD8 naive | 9.29 | 0.55 |
| NK | 9.02 | 0.64 |
| Unconventional T cells | 6.27 | 0.33 |
| Non-classical monocytes | 2.06 | 0.69 |
| pDC | 0.46 | 0.82 |

**Table S2.** Silhouette Scores for the Kotliarov2020 [35] dataset for each cell type in their broad cell type annotations.

| Cell Type (fine) | Proportion (%) | Silhouette Score |
|---|---|---|
| CD4 naive | 20.94 | 0.61 |
| CD4 central and transitional memory T | 15.75 | 0.34 |
| Classical monocytes | 12.08 | 0.32 |
| CD8 naive | 9.29 | 0.54 |
| CD16++ NK | 8.59 | 0.52 |
| Transitional B cells | 8.11 | 0.16 |
| CD8 central and transitional memory T | 4.77 | -0.11 |
| CD8 effector memory and temra | 4.36 | 0.06 |
| CD161 double-negative T | 3.61 | -0.06 |
| Unconventional CD161h/CD8+ T | 2.66 | 0.39 |
| Non-classical monocytes | 2.08 | 0.65 |
| CD4 effector memory and temra | 1.85 | 0.35 |
| Switched B cells | 1.59 | 0.12 |
| Unswitched B cells | 1.45 | 0.20 |
| NKT-like (CD56+/CD3+) | 0.65 | 0.19 |
| pDC | 0.46 | 0.82 |
| CD56h/CD16l NK | 0.42 | -0.13 |
| Double-negative T | 0.39 | -0.10 |
| CD8+ CD103+ T | 0.31 | 0.37 |
| IgA monocytes | 0.26 | 0.01 |
| mDC | 0.25 | 0.32 |
| HSC | 0.11 | -0.20 |

**Table S3.** Silhouette Scores for the Kotliarov2020 [35] dataset for each cell type in their fine (level 3) cell type annotations.

| Cell Type | Proportion (%) | Silhouette Score |
|---|---|---|
| Mono | 30.297223 | 0.567191 |
| CD4 T | 25.346183 | 0.471439 |
| CD8 T | 15.744541 | 0.049871 |
| NK | 11.537796 | 0.472371 |
| B | 8.530946 | 0.718609 |
| other T | 4.196855 | 0.072382 |
| DC | 2.218664 | 0.200626 |
| other | 2.127791 | -0.022007 |

**Table S4.** Silhouette Scores for the Hao2021 [68] dataset for each cell type in their broad (level 1) cell type annotations.

| Cell Type | Proportion (%) | Silhouette Score |
|---|---|---|
| CD14 Mono | 26.39 | 0.29 |
| CD4 Naive | 10.81 | 0.17 |
| NK | 10.62 | 0.28 |
| CD4 TCM | 9.20 | 0.06 |
| CD8 TEM | 7.25 | 0.02 |
| CD8 Naive | 6.66 | 0.38 |
| B naive | 4.77 | 0.22 |
| CD16 Mono | 3.91 | 0.38 |
| CD4 TEM | 2.65 | 0.03 |
| gdT | 2.26 | -0.20 |
| B memory | 2.03 | 0.17 |
| CD8 TCM | 1.78 | -0.05 |
| MAIT | 1.72 | 0.40 |
| Treg | 1.55 | 0.14 |
| cDC2 | 1.55 | 0.26 |
| B intermediate | 1.50 | 0.01 |
| Platelet | 1.42 | 0.37 |
| CD4 CTL | 1.07 | 0.25 |
| NK_CD56bright | 0.58 | 0.29 |
| pDC | 0.53 | 0.47 |
| Doublet | 0.37 | -0.39 |
| NK Proliferating | 0.34 | -0.10 |
| Plasmablast | 0.23 | 0.39 |
| dnT | 0.22 | -0.28 |
| HSPC | 0.20 | -0.01 |
| cDC1 | 0.09 | 0.33 |
| ILC | 0.08 | 0.11 |
| CD4 Proliferating | 0.07 | -0.18 |
| CD8 Proliferating | 0.06 | 0.22 |
| Eryth | 0.05 | 0.19 |
| ASDC | 0.05 | 0.21 |

**Table S5.** Silhouette Scores for the Hao2021 [68] dataset for each cell type in their fine (level 2) cell type annotations.

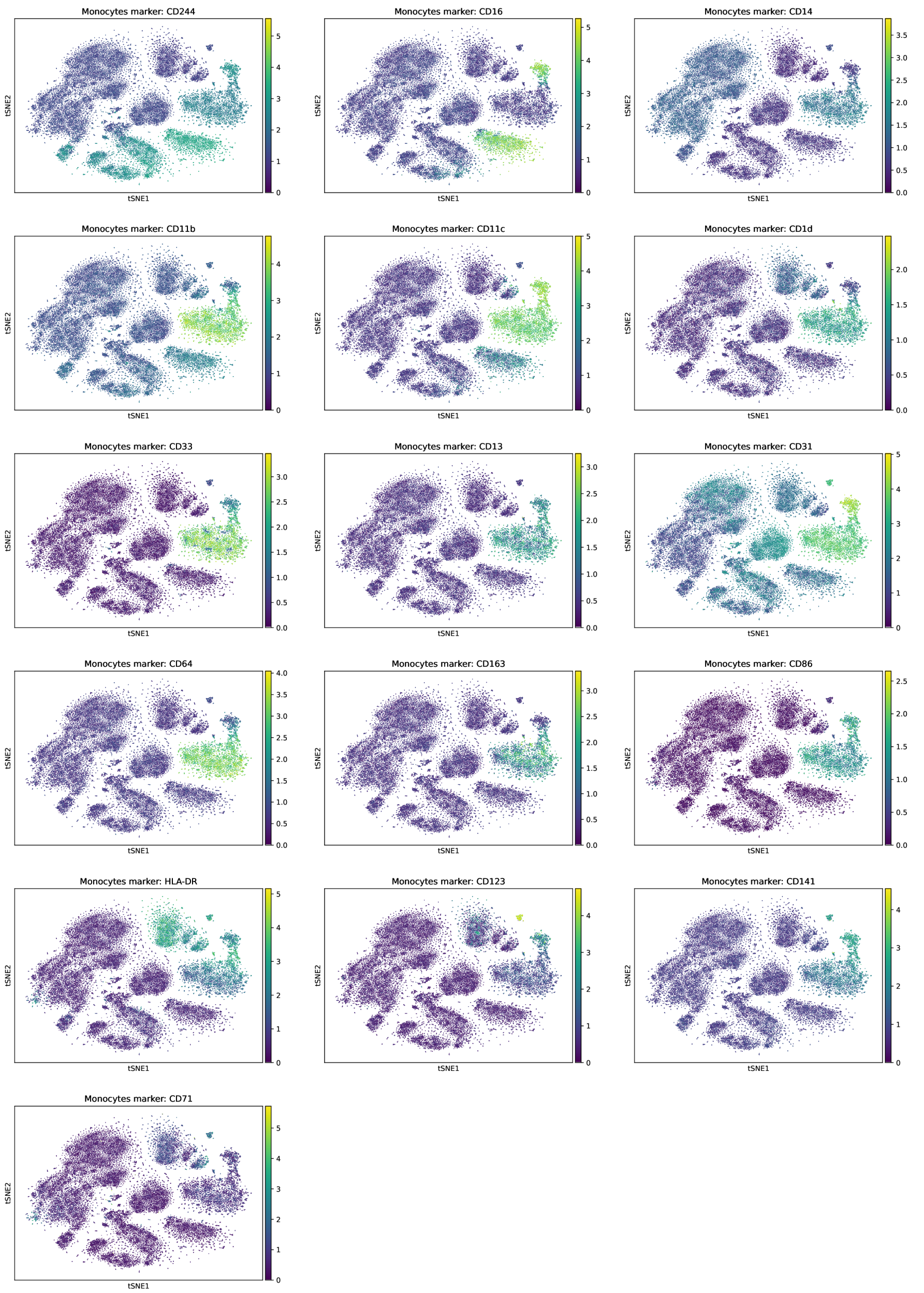


**Fig. S2.** A t-SNE [46] visualisation of one of our multi-omics model's learned latent representation of the [35] dataset. We overlay the $\log_{10}$ surface-protein expression for the marker proteins of Monocytes defined by [35].

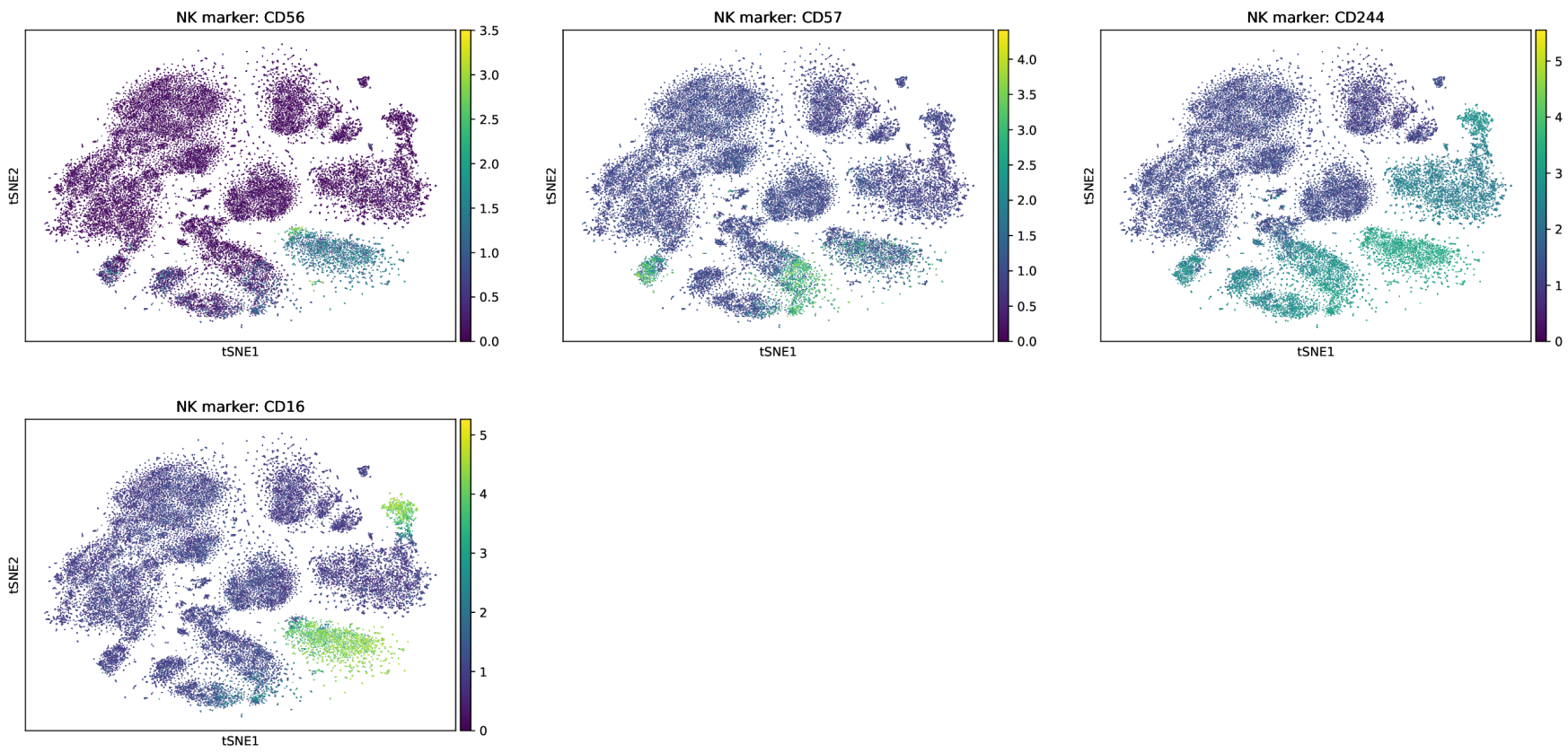


**Fig. S3.** A t-SNE [46] visualisation of one of our multi-omics model's learned latent representation of the [35] dataset. We overlay the $\log_{10}$ surface-protein expression for the marker proteins of Natural Killer (NK) cells defined by [35].

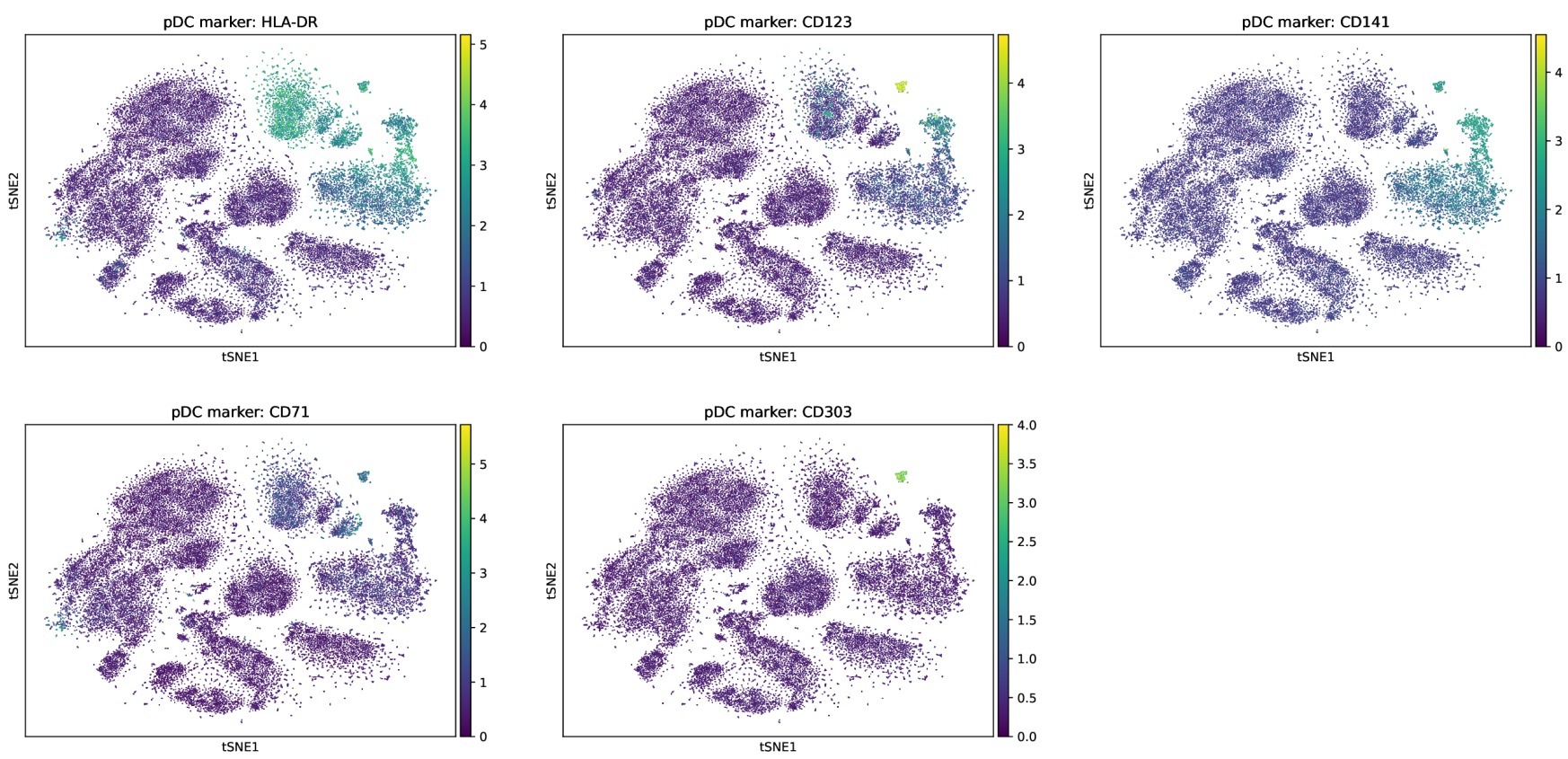


**Fig. S4.** A t-SNE [46] visualisation of one of our multi-omics model's learned latent representation of the [35] dataset. We overlay the $\log_{10}$ surface-protein expression for the marker proteins of pDC cells defined by [35].

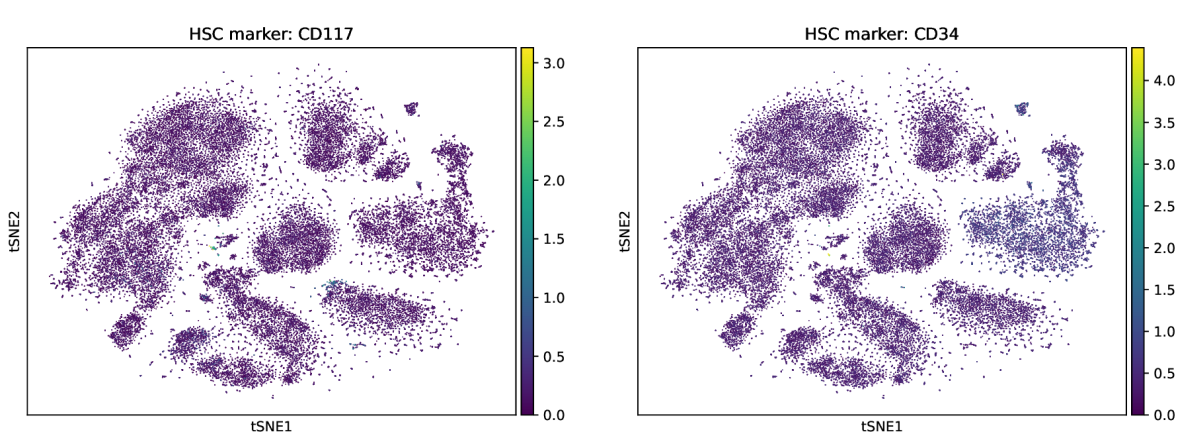


**Fig. S5.** A t-SNE [46] visualisation of one of our multi-omics model's learned latent representation of the [35] dataset. We overlay the $\log_{10}$ surface-protein expression for the marker proteins of HSC cells defined by [35].

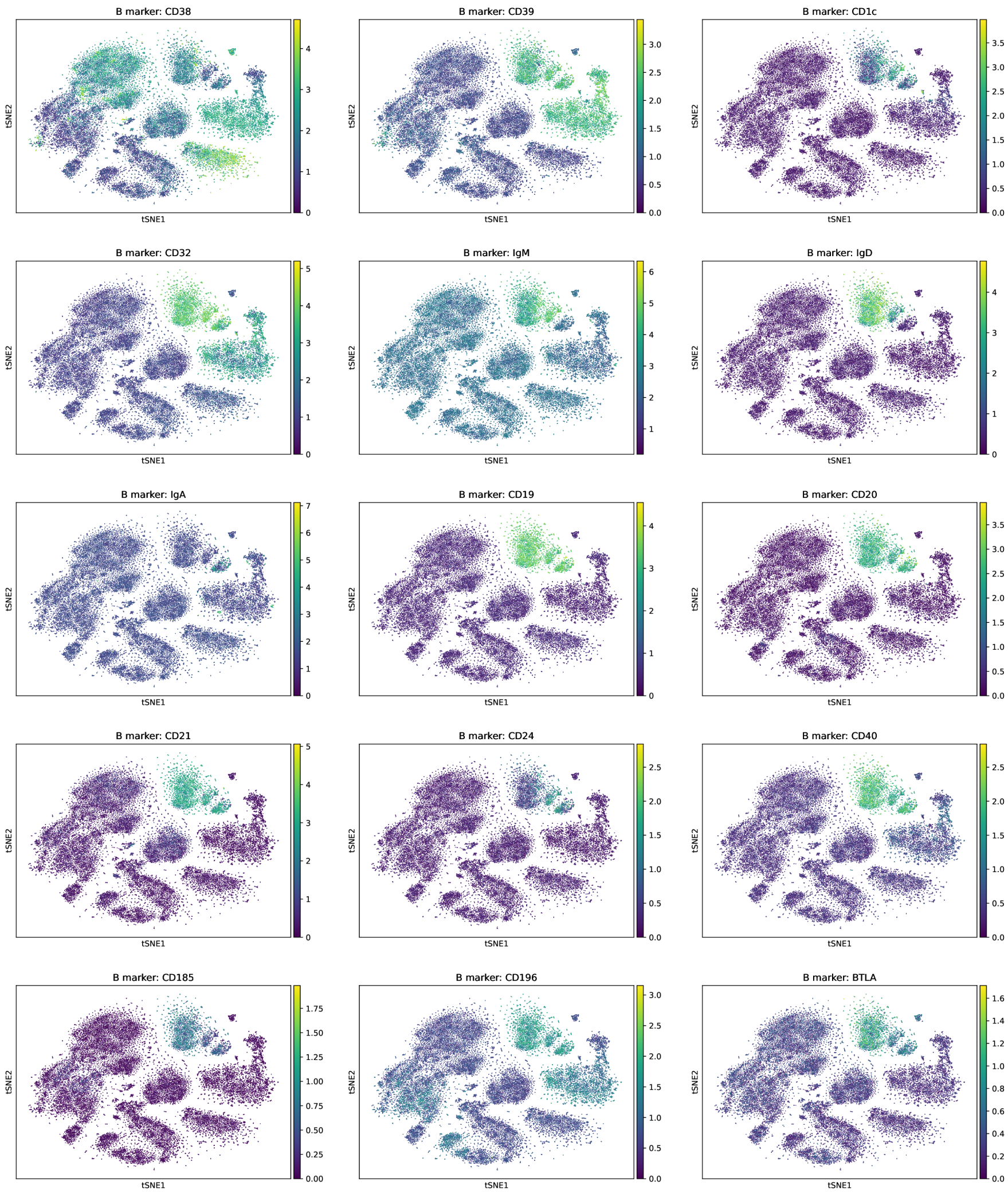


**Fig. S6.** A t-SNE [46] visualisation of one of our multi-omics model's learned latent representation of the [35] dataset. We overlay the $\log_{10}$ surface-protein expression for the marker proteins of B-cells defined by [35].

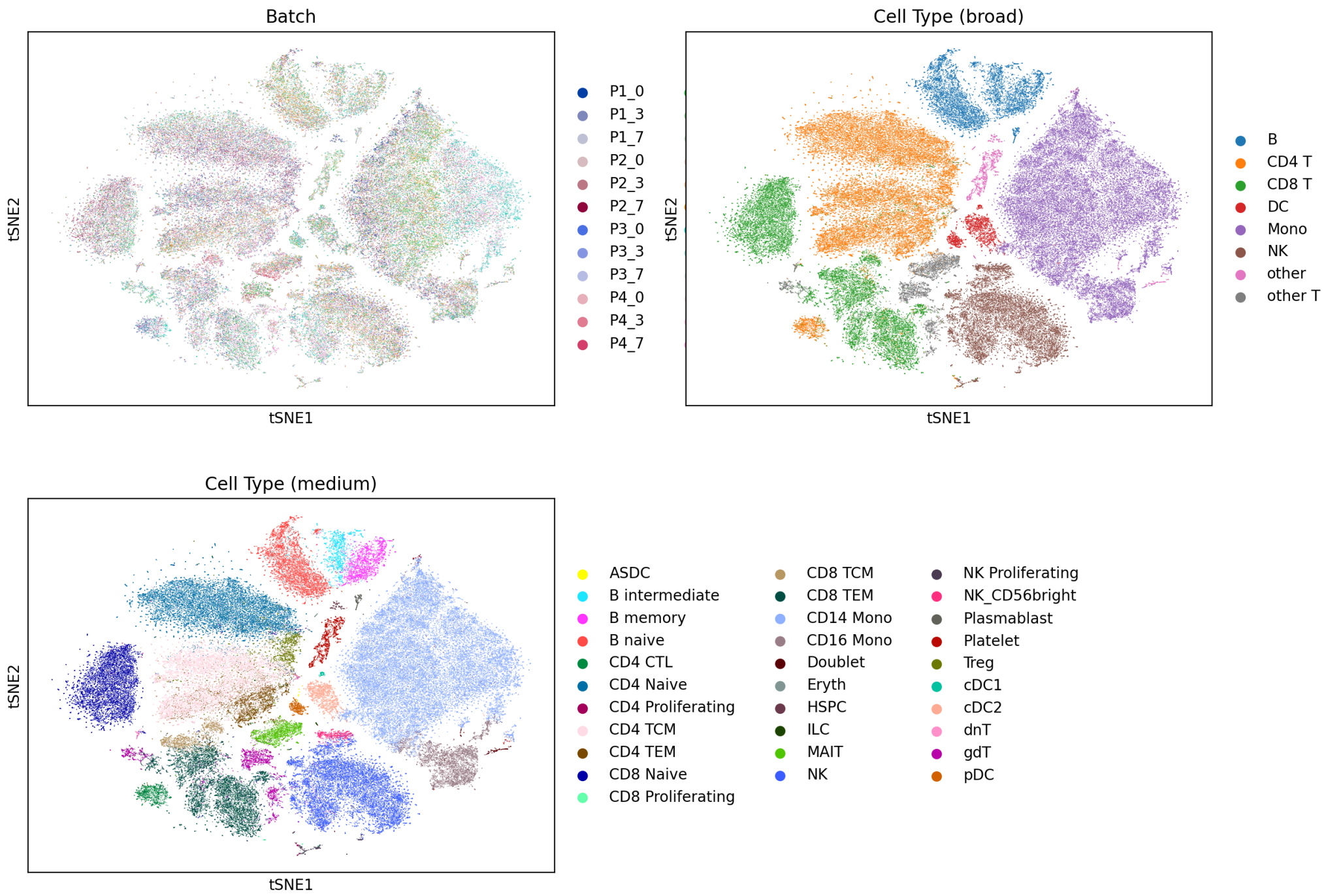


**Fig. S7.** TSNE visualisation of the resulting SCONE embedding on the Hao2021 dataset.

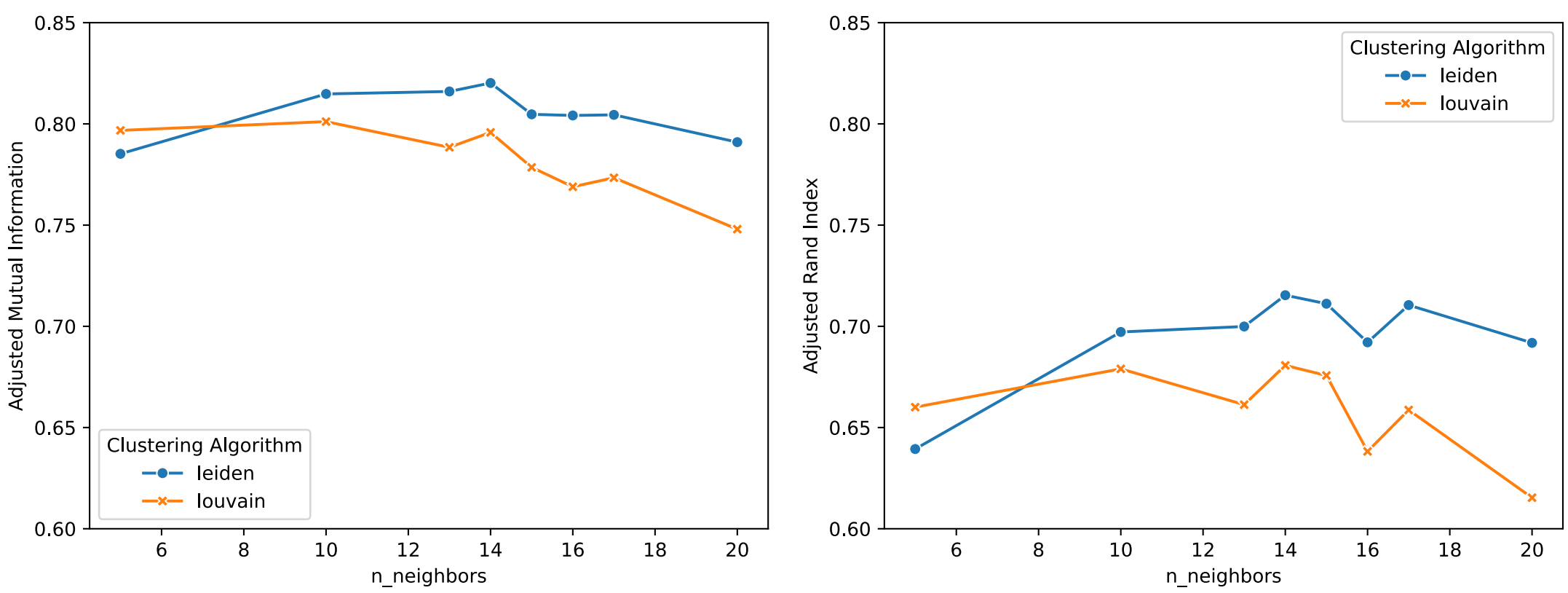


**Fig. S8.** Plots on the Kotliarov2020 dataset showing the average Adjusted Mutual Information (left) and Adjusted Rand Index (right) over 4 runs while varying the number of neighbours. Results are shown for both Leiden and Louvain clustering, demonstrating relatively stable performance across a range of neighbourhood sizes. However, for larger *k* values the performance under the louvain algorithm seems to be in a slight downward trajectory, we hypothesise that this might be due to reduced local cluster separability due to denser neighbourhood graphs.

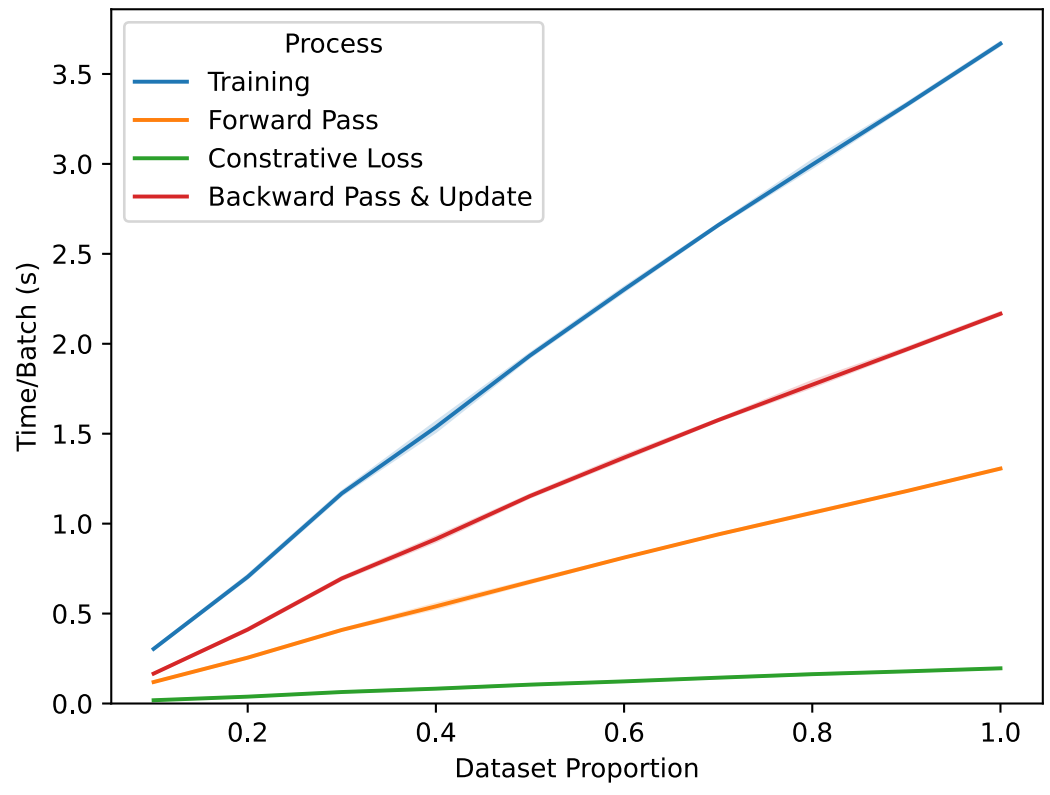


**Fig. S9.** Time taken for the forward pass, calculating the contrastive loss, and backward pass and update per batch, as well as the total training time, by varying the input dataset size and having subsets of 20% of the dataset. Shaded area represents the standard deviation across runs. Model ran on the Kotliarov2020 dataset, with 16 repetitions, using the CPU.

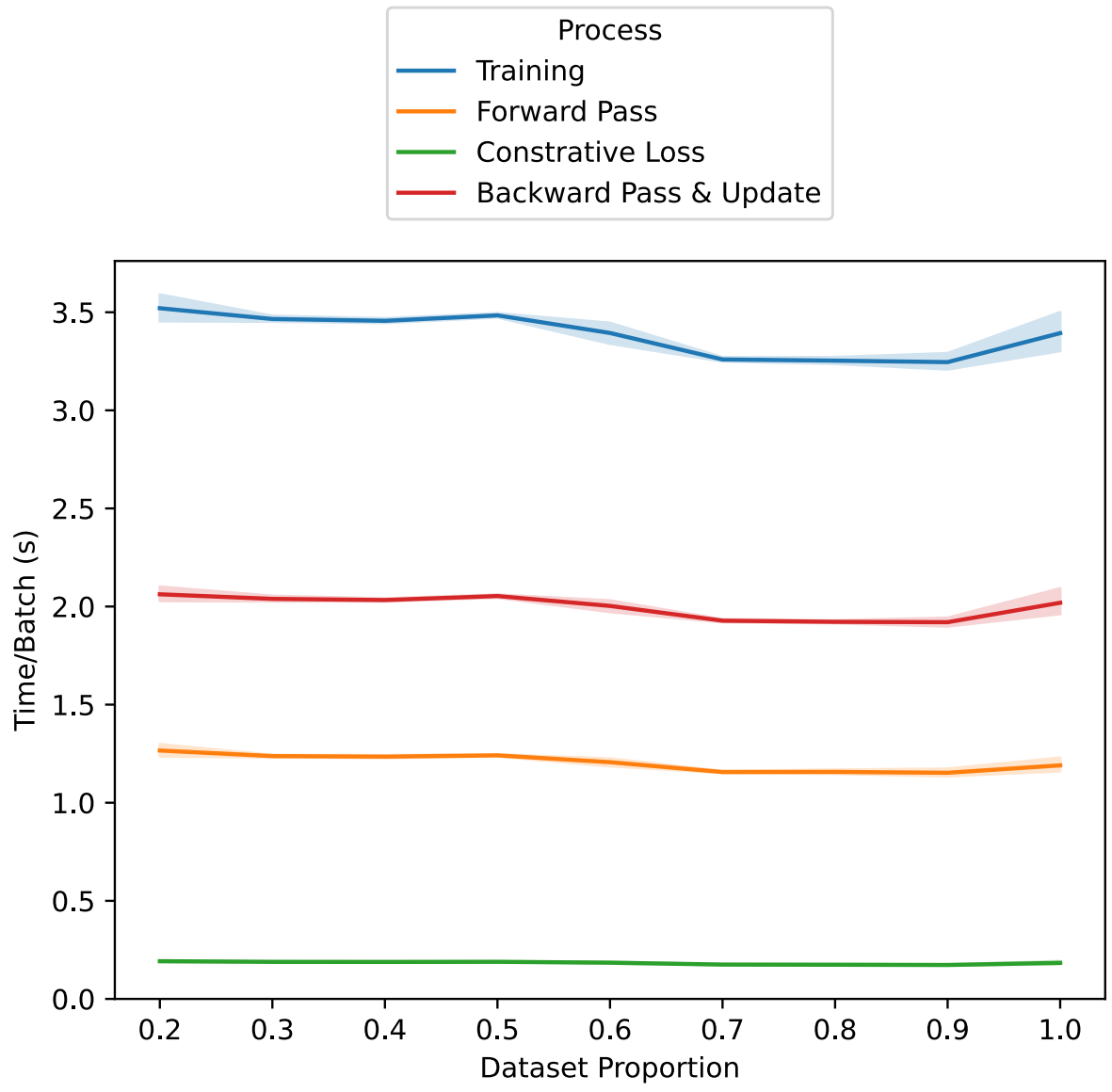


**Fig. S10.** Time taken for the forward pass, calculating the contrastive loss, and backward pass and update per batch, as well as the total training time, by keeping fixed the number of samples that make up each subset, and varying the dataset size. Note that, while the time per batch remains constant, as the dataset size increases, we would recommend a sufficient amount of batches to be ran so that probabilistically the whole dataset is covered. Shaded area represents the standard deviation across runs. Model ran on the Kotliarov2020 dataset, with 16 repetitions, using the CPU.

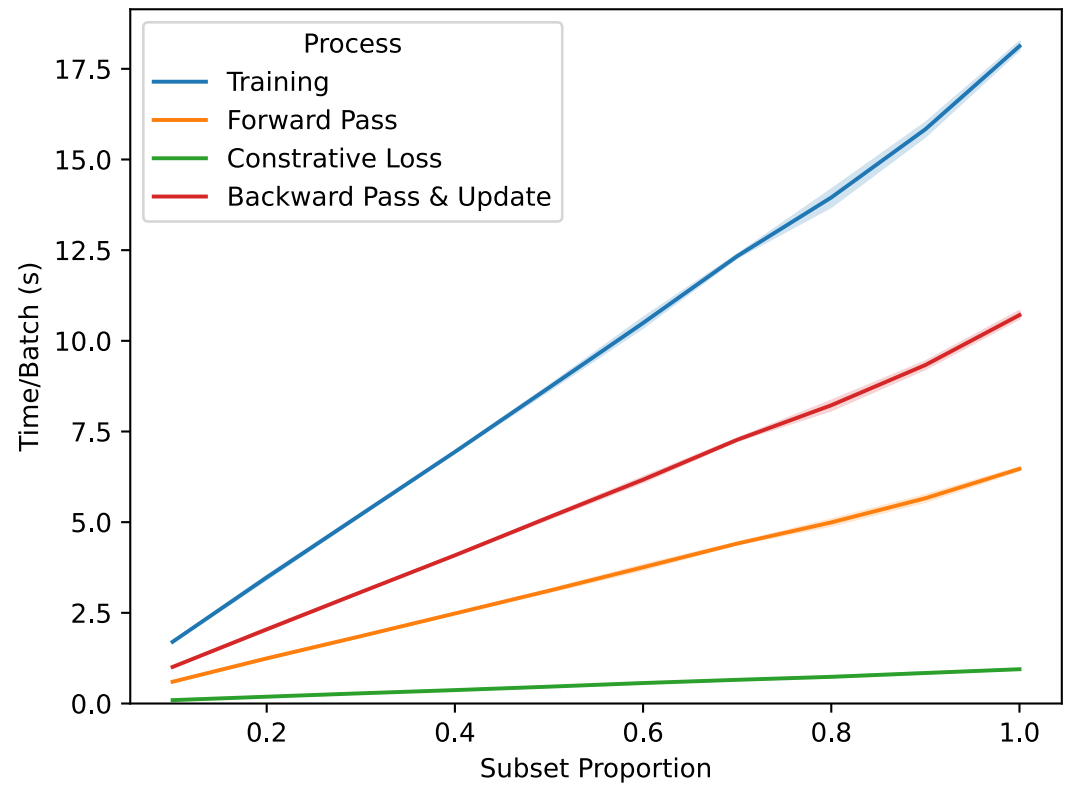


**Fig. S11.** Time taken for the forward pass, calculating the contrastive loss, and backward pass and update per batch, as well as the total training time, by keeping fixed the dataset size, and varying the proportion of the dataset size used for the subset. Note that, while the time scales linearly with dataset size, in practice we would recommend a sufficient amount of batches to be ran so that probabilistically the whole dataset is covered. Shaded area represents the standard deviation across runs. Model ran on the Kotliarov2020 dataset, with 16 repetitions, using the CPU.

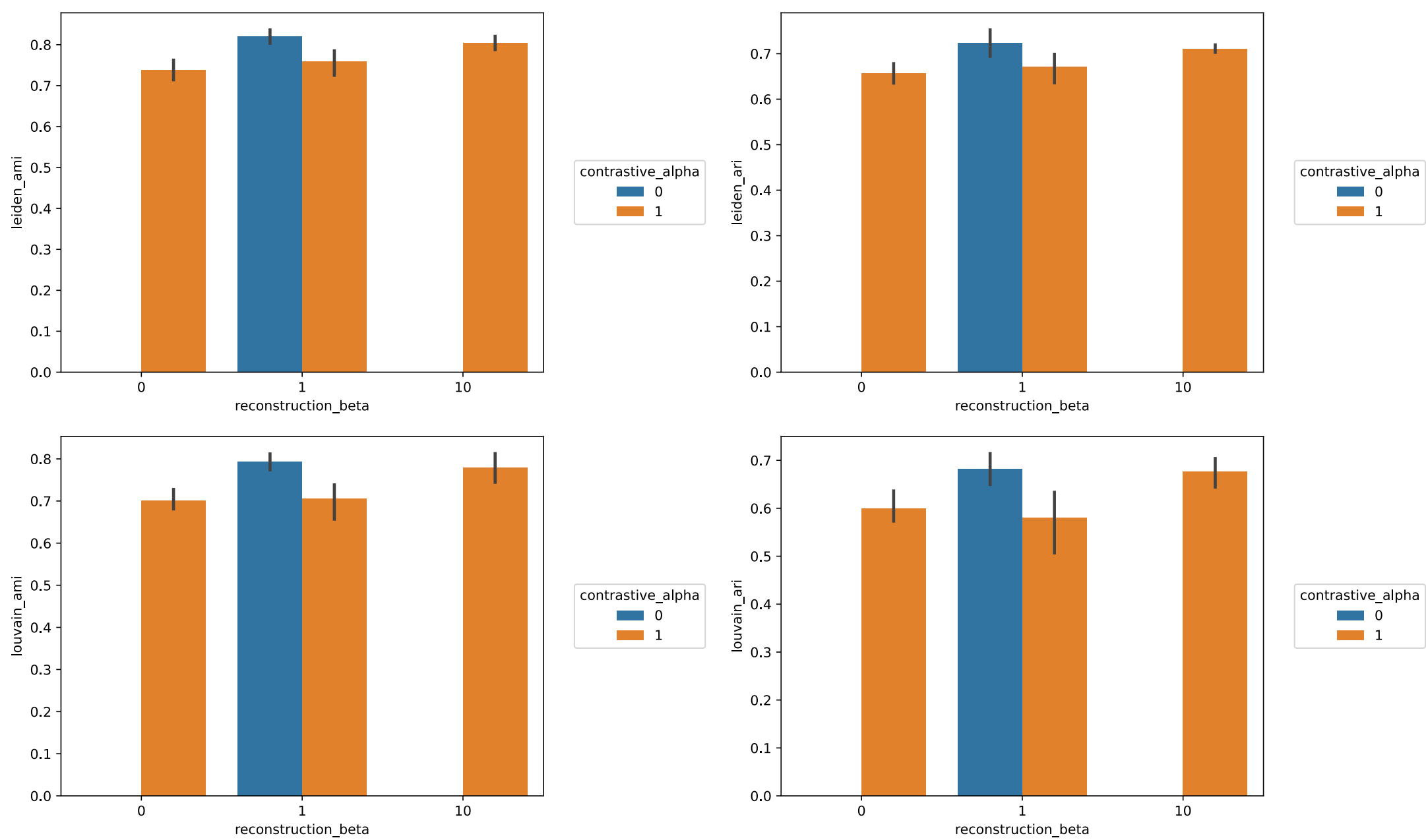


**Fig. S12.** An ablation on the Korliarov2020 dataset showing the average performance over 4 runs varying the loss weighting hyperparameters. Note that this plot was produced during the review process and did not influence the hyperparameter selection for comparison purposes. The difference between only reconstructing the input ($\alpha = 0, \beta = 1$) and our chosen hyperparameters ($\alpha = 1, \beta = 10$) was not statistically significant, and had been selected on preliminary studies on other data, where aligning the views provided qualitatively better results.
